# JAK1/2 inhibition with baricitinib in the treatment of autoinflammatory interferonopathies

**DOI:** 10.1172/JCI98814

**Published:** 2018-06-11

**Authors:** Gina A. Montealegre Sanchez, Adam Reinhardt, Suzanne Ramsey, Helmut Wittkowski, Philip J. Hashkes, Yackov Berkun, Susanne Schalm, Sara Murias, Jason A. Dare, Diane Brown, Deborah L. Stone, Ling Gao, Thomas Klausmeier, Dirk Foell, Adriana A. de Jesus, Dawn C. Chapelle, Hanna Kim, Samantha Dill, Robert A. Colbert, Laura Failla, Bahar Kost, Michelle O’Brien, James C. Reynolds, Les R. Folio, Katherine R. Calvo, Scott M. Paul, Nargues Weir, Alessandra Brofferio, Ariane Soldatos, Angelique Biancotto, Edward W. Cowen, John J. Digiovanna, Massimo Gadina, Andrew J. Lipton, Colleen Hadigan, Steven M. Holland, Joseph Fontana, Ahmad S. Alawad, Rebecca J. Brown, Kristina I. Rother, Theo Heller, Kristina M. Brooks, Parag Kumar, Stephen R. Brooks, Meryl Waldman, Harsharan K. Singh, Volker Nickeleit, Maria Silk, Apurva Prakash, Jonathan M. Janes, Seza Ozen, Paul G. Wakim, Paul A. Brogan, William L. Macias, Raphaela Goldbach-Mansky

**Affiliations:** 1 Translational Autoinflammatory Disease Section, National Institute of Allergy and Infectious Diseases (NIAID), NIH, Bethesda, Maryland, USA.; 2 Faculty of Physicians of the University of Nebraska Medical Center, College of Medicine, Omaha, Nebraska, USA.; 3 IWK Health Centre, Halifax, Nova Scotia, Canada.; 4 Department of Pediatric Rheumatology and Immunology, University Children’s Hospital, Muenster, Germany.; 5 Shaare-Zedek Medical Center, Jerusalem, Israel.; 6 Hadassah Hebrew University Medical Center, Jerusalem, Israel.; 7 Hauner Children’s Hospital LMU, Munich, Germany.; 8 Hospital Infantil La Paz, Madrid, Spain.; 9 University of Arkansas for Medical Sciences, Little Rock, Arkansas, USA.; 10 Children’s Hospital Los Angeles, Los Angeles, California, USA.; 11 National Human Genome Research Institute, NIH, Bethesda, Maryland, USA.; 12 Riley Hospital for Children, Indianapolis, Indiana, USA.; 13 National Institute of Arthritis and Musculoskeletal and Skin Diseases (NIAMS), NIH, Bethesda, Maryland, USA.; 14 Clinical Center, NIH, Bethesda, Maryland, USA.; 15 National Heart, Lung, and Blood Institute (NHLBI), NIH, Bethesda, Maryland, USA.; 16 National Institute of Neurological Disorders and Stroke (NINDS), NIH, Bethesda, Maryland, USA.; 17 National Cancer Institute (NCI), NIH, Bethesda, Maryland, USA.; 18 Walter Reed National Military Medical Center, Bethesda, Maryland, USA.; 19 NIAID, NIH, Bethesda, Maryland, USA.; 20 National Institute of Diabetes and Digestive and Kidney Diseases (NIDDK), NIH, Bethesda, Maryland, USA.; 21 University of North Carolina School of Medicine, Chapel Hill, North Carolina, USA.; 22 Eli Lilly and Company, Indianapolis, Indiana, USA.; 23 Hacettepe University Faculty of Medicine, Ankara, Turkey.; 24 Biostatistics and Clinical Epidemiology Service, NIH Clinical Center, Bethesda, Maryland, USA.; 25 University College London (UCL) Great Ormond Street Institute of Child Health and Great Ormond Street Hospital NHS Foundation, London, United Kingdom.

**Keywords:** Immunology, Therapeutics, Innate immunity, Monogenic diseases, Translation

## Abstract

**BACKGROUND.** Monogenic IFN–mediated autoinflammatory diseases present in infancy with systemic inflammation, an IFN response gene signature, inflammatory organ damage, and high mortality. We used the JAK inhibitor baricitinib, with IFN-blocking activity in vitro, to ameliorate disease.

**METHODS.** Between October 2011 and February 2017, 10 patients with CANDLE (chronic atypical neutrophilic dermatosis with lipodystrophy and elevated temperatures), 4 patients with SAVI (stimulator of IFN genes–associated [STING-associated] vasculopathy with onset in infancy), and 4 patients with other interferonopathies were enrolled in an expanded access program. The patients underwent dose escalation, and the benefit was assessed by reductions in daily disease symptoms and corticosteroid requirement. Quality of life, organ inflammation, changes in IFN-induced biomarkers, and safety were longitudinally assessed.

**RESULTS.** Eighteen patients were treated for a mean duration of 3.0 years (1.5–4.9 years). The median daily symptom score decreased from 1.3 (interquartile range [IQR], 0.93–1.78) to 0.25 (IQR, 0.1–0.63) (*P* < 0.0001). In 14 patients receiving corticosteroids at baseline, daily prednisone doses decreased from 0.44 mg/kg/day (IQR, 0.31–1.09) to 0.11 mg/kg/day (IQR, 0.02–0.24) (*P* < 0.01), and 5 of 10 patients with CANDLE achieved lasting clinical remission. The patients’ quality of life and height and bone mineral density *Z*-scores significantly improved, and their IFN biomarkers decreased. Three patients, two of whom had genetically undefined conditions, discontinued treatment because of lack of efficacy, and one CANDLE patient discontinued treatment because of BK viremia and azotemia. The most common adverse events were upper respiratory infections, gastroenteritis, and BK viruria and viremia.

**CONCLUSION.** Upon baricitinib treatment, clinical manifestations and inflammatory and IFN biomarkers improved in patients with the monogenic interferonopathies CANDLE, SAVI, and other interferonopathies. Monitoring safety and efficacy is important in benefit-risk assessment.

**TRIAL REGISTRATION.** ClinicalTrials.gov NCT01724580 and NCT02974595.

**FUNDING.** This research was supported by the Intramural Research Program of the NIH, NIAID, and NIAMS. Baricitinib was provided by Eli Lilly and Company, which is the sponsor of the expanded access program for this drug.

## Introduction

The IFN-mediated autoinflammatory diseases CANDLE (chronic atypical neutrophilic dermatosis with lipodystrophy and elevated temperatures) and SAVI (stimulator of IFN genes–associated [STING-associated] vasculopathy with onset in infancy) are Mendelian innate immune–dysregulatory disorders that present early in life with fevers, sterile organ inflammation, and a high type I IFN response gene signature (IRS) in peripheral blood cells (1, 2) and are part of the spectrum of conditions termed interferonopathies (3). CANDLE is caused by loss-of-function mutations in genes encoding proteasome complexes that regulate protein degradation (4–8). Patients with CANDLE present with fever, neutrophilic panniculitis, lipodystrophy, cytopenias, myositis, and lymphocytic aseptic meningitis. Forty to eighty percent of patients develop systemic hypertension, metabolic syndrome, and hepatic steatosis, often in the first decade of life (2). SAVI is caused by gain-of-function mutations in the viral sensor STING, resulting in constitutive transcription of the potent antiviral cytokine IFNβ (9–11). Patients present with cold-induced acral vasculitis resulting in loss of digits and interstitial lung disease, the latter of which may be the presenting symptom (2, 9).

Both syndromes respond poorly to biologic disease–modifying antirheumatic drugs (DMARDs) that target proinflammatory cytokines (i.e., IL-1, TNF, and IL-6) (8, 9) or to conventional DMARDs. A high IRS is absent in patients with clinically active autoinflammatory diseases who respond to treatment with IL-1–blocking agents (9, 12). Together, these findings support a potential role for type I IFN in propagating systemic organ inflammation and damage and high mortality rates (2).

Until recently, treatments that block IFN signaling have not been available. However, the JAK/STAT pathway constitutes the principal signaling pathway for cytokine and growth factor receptors including the IFN-α/β receptor (IFNAR) and the IFN-γ receptor (IFNGR) (13, 14). Small molecules that inhibit JAKs reduce type I and type II IFN–induced STAT1 phosphorylation (p-STAT1) in CANDLE and SAVI patients in vitro (7, 9), which suggested their potential utility in reducing the IFN signaling and disease manifestations in these patients. In October 2011, we developed an expanded access program with baricitinib, a selective JAK1 and JAK2 inhibitor (15) that is currently approved for the treatment of moderately to severely active rheumatoid arthritis (RA) in adults (16) in over 40 countries including European countries, Japan, and and the United States, to treat patients with CANDLE, SAVI, and other presumed interferonopathies. Data were collected to ensure patient safety and assess benefit and to determine whether continuation of baricitinib administration was justified.

## Results

### Clinical manifestations of CANDLE, SAVI, and other interferonopathies.

Between October 2011 and October 2016, we treated 18 patients, 10 with genetically confirmed CANDLE, 4 with genetically confirmed SAVI, and 4 patients with other interferonopathies. One patient was later found to have Aicardi Goutières syndrome 5 (AGS5), and one had a novel disease-causing mutation. The baseline demographics and clinical characteristics are summarized in Table 1. All of the CANDLE and SAVI patients developed disease symptoms in the first 2.5 weeks of life. The mean age at enrollment was 12.5 years (range, 1.2–24.1); 72% of patients were below the third percentile for height; and 50% of patients were below the third percentile for weight (Table 1). Fourteen of eighteen (78%) patients were on chronic corticosteroid treatment for an average of 5.7 years (range, 1–17 years) prior to entry into the program. Three patients with SAVI and one patient with CANDLE had failed and discontinued corticosteroids prior to enrollment. All patients had failed in the use of 1 to 6 conventional and/or biologic DMARDs. Most patients had frequent and prolonged hospitalizations prior to enrollment.

### Clinical symptoms improve upon treatment with baricitinib.

**Figure 1:**
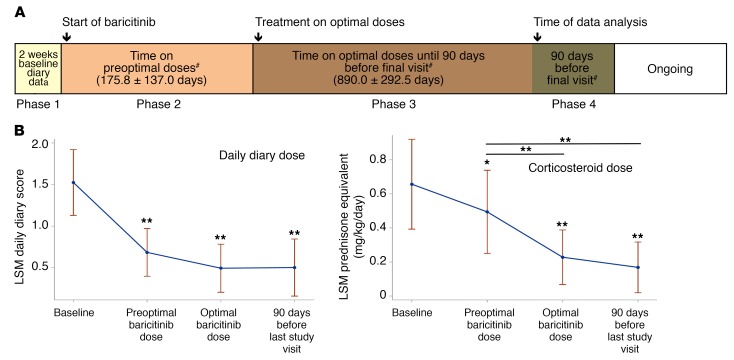
Expanded access program overview and effect of baricitinib treatment on clinical outcomes. (**A**) Expanded access program overview. Phase 1: Time before the first baricitinib dose. Phase 2: Period of dose escalation, including the time between the first baricitinib dose and achievement of an optimal dose regimen. Phase 3: Time on optimal baricitinib doses, excluding the last 90 days prior to the final visit. Phase 4: Ninety days prior to the final visit, with analysis of primary data (daily diaries, steroid doses, and biomarkers of IFN signaling). The program is ongoing. ^#^The number of days in each phase is reported as the mean ± SD. For phases 2 and 3, patients O1 and O3 were not included in the calculation. Both patients discontinued treatment because of a lack of efficacy and/or osteonecrosis after only 77 and 56 days on optimal doses, respectively. (B) Effect of baricitinib treatment on clinical outcomes. To confirm trends in longitudinally collected data, the diary scores and corticosteroid doses were fitted to a repeated-measures model with “phase” as a categorical independent variable. Least-squares means with 95% CIs for each phase were assessed. *P < 0.05 and **P < 0.001 (both unadjusted) by 2-sided paired Student’s *t* test.

**Figure 2:**
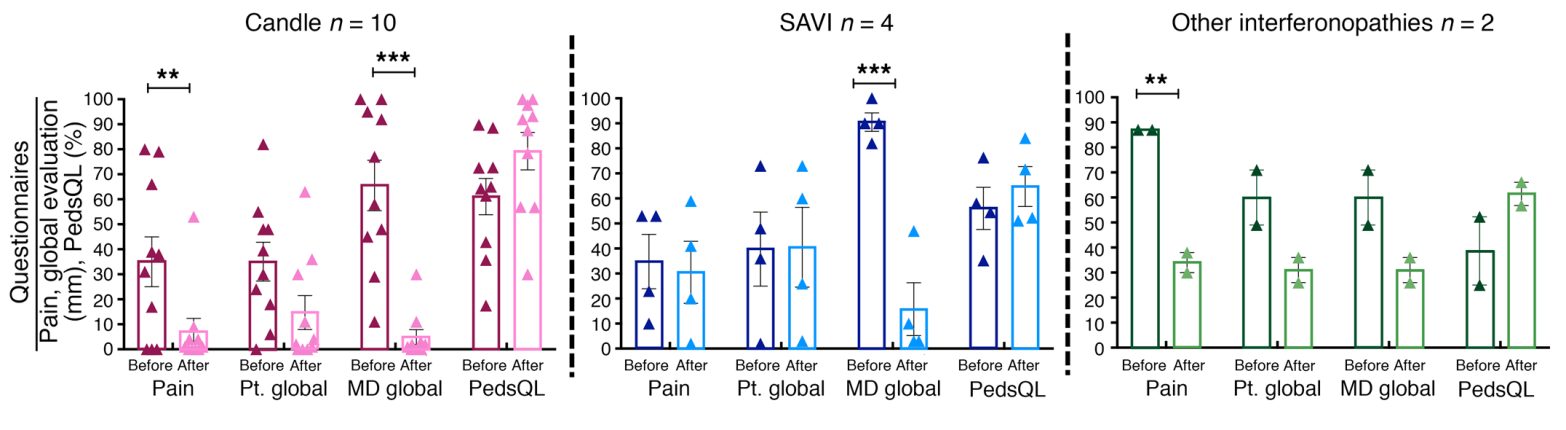
Self-reported and physician’s global assessments by disease subgroup. Parent’s or patient’s overall assessment of pain and health (Pt. global) and the physician’s assessment (MD global) were measured using a visual analog scale (VAS), in which a value of 100 mm indicates the worst possible measure for the condition assessed by the test. Quality of life (PedsQL) was measured using a standardized age-matched test that ranged from 0% to 100%, with higher percentages indicating improvement. Data are presented by disease, with CANDLE in red, SAVI in blue, and other interferonopathies in green. Only the 2 patients who stayed in the study are shown. Darker shades indicate pretreatment, and lighter shades indicate the last included visit on baricitinib treatment. **P < 0.05 and ***P < 0.001 (both unadjusted) by 2-sided paired Student’s *t* test.

**Figure 3:**
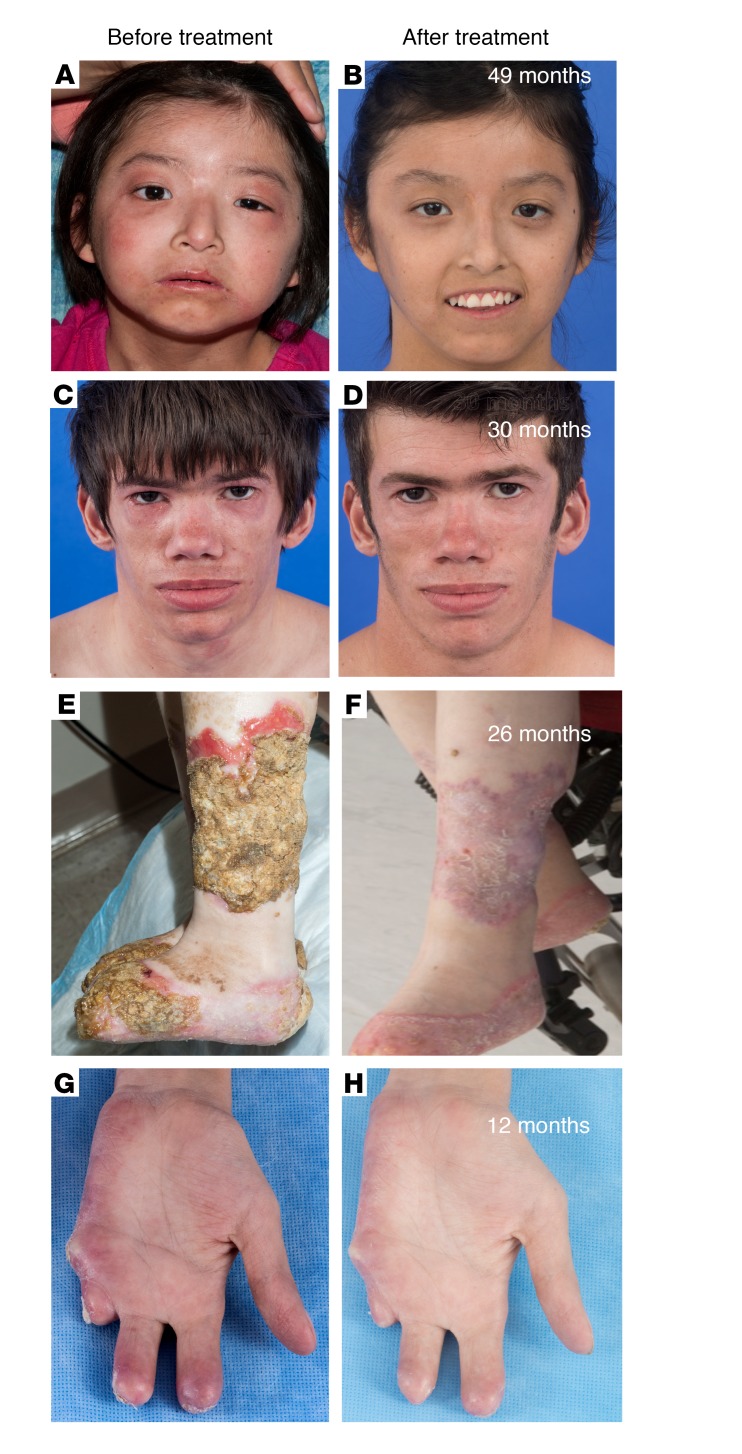
Improvement in clinical disease manifestations in CANDLE and SAVI patients treated with baricitinib. (**A**–**D**) Images of 2 patients with CANDLE who achieved the remission criteria (C2 and C10, respectively) are shown. Pretreatment images of the face show typical distribution of facial panniculitis with periorbital swelling and erythema as well as lipodystrophy affecting temporal regions and areas above and below the zygomatic bone. Lip swelling is also evident. Post-treatment images show complete resolution of areas of panniculitis on the face and neck. (**E** and **F**) Images of 2 of the 4 patients with SAVI are shown. Images of the lower leg of a SAVI patient (S3) show extensive eschar formation overlying infected, nonhealing ulcers on the left lower leg. After treatment, the ulcers healed, with complete reepithelialization. (**G** and **H**) Images of the right palmar surface of the hand of a patient with SAVI (S4) show chronic cutaneous vasculitis that resulted in partial amputation of the second and third fingers and complete loss of the fourth and fifth fingers. On baricitinib treatment, a significant improvement in cutaneous vasculitis resulted in preservation of the fingers without further tissue loss.

All patients underwent dose escalation until they reached optimal tolerated treatment doses (Figure 1A). The median duration in the program at the time of analysis was 1,023 days, or 2.8 years (IQR, 842–1,419.5); patients had been on optimized doses for a median of 897 days, or 2.5 years (IQR, 639–1,160 days) (Supplemental Table 1; supplemental material available online with this article; https://doi.org/10.1172/JCI98814DS1). At the last NIH visit, 12 of 18 (67%) patients fulfilled the diary score improvement criteria (80% of CANDLE, 75% of SAVI, and 1 of 4 [25%] patients with other interferonopathies). Of the 14 patients on corticosteroids at baseline, 10 of 14 (71%) fulfilled the corticosteroid improvement criteria (Table 2 and Supplemental Table 3). The median diary score decreased from 1.3 (IQR, 0.93–1.78) at baseline to 0.25 (IQR, 0.10–0.63) (*P* < 0.0001). The median corticosteroid dose dropped from a prednisone equivalent dose of 0.44 (IQR, 0.31–1.09) mg/kg/day at baseline to 0.11 (IQR, 0.02–0.24) mg/kg/day (*P* < 0.005) (Table 3). All available data were used in a repeated-measures model to assess responses over time. Least-squares means for diary scores and corticosteroid dose decreased from baseline (phase 1), during the baricitinib dose escalation (phase 2), and further when optimal treatment doses were reached (phase 3), and remained stable during the last 90 days of the observation period (phase 4). Patients were weaned from corticosteroids during dose escalation and were further weaned on optimal tolerated baricitinib doses (*P* < 0.001 for both, respectively, Figure 1B). Patient pain, overall wellbeing, and quality of life improved with treatment (Figure 2): 5 (50%) patients with CANDLE achieved remission with no disease symptoms (disease-specific daily symptom score [DDS] <0.15) and normal C-reactive protein (CRP), despite discontinuation of corticosteroids (Table 2). The patients’ CRP was below 5 mg/l in 84.6% of the subsequent visits, and the IFN response gene scores were normal in 66.7% of visits at the last follow-up, which encompassed a mean of 654.4 (range, 581–822) days after the patients first achieved remission criteria until data analysis, suggesting durable remission (Supplemental Table 4). The clinical responses were most pronounced in patients with CANDLE, while in patients with SAVI, the vasculitis flares improved but still occurred, albeit with reduced duration and severity; none of the SAVI patients experienced further loss of digits (Figure 3, A–H and Supplemental Figure 2, A and B). The patient with SAVI on corticosteroid treatment at baseline had an initial reduction but increased corticosteroid doses prior to the final visit because of subjective symptoms of respiratory difficulties. In the context of stable pulmonary function tests (PFTs) and chest CT, her corticosteroid dose was subsequently reduced to 0.11 mg/kg/day.

Three patients discontinued treatment. Two patients without a genetic diagnosis stopped after 244 and 98 days of treatment, respectively one because lack of efficacy and the other because of osteonecrosis and an unsatisfactory treatment response. One CANDLE patient, for whom the corticosteroid dose could not be tapered, developed BK viremia and azotemia and discontinued treatment (Supplemental Figure 1). The 2 patients with other interferonopathies, both of whom stayed on treatment (1 patient with AGS5 and 1 with a novel disease-causing mutation), had symptom improvements and lowered their corticosteroid dose to less than 0.15 mg/kg/day (Supplemental Figure 2, C–H).

**Figure 4:**
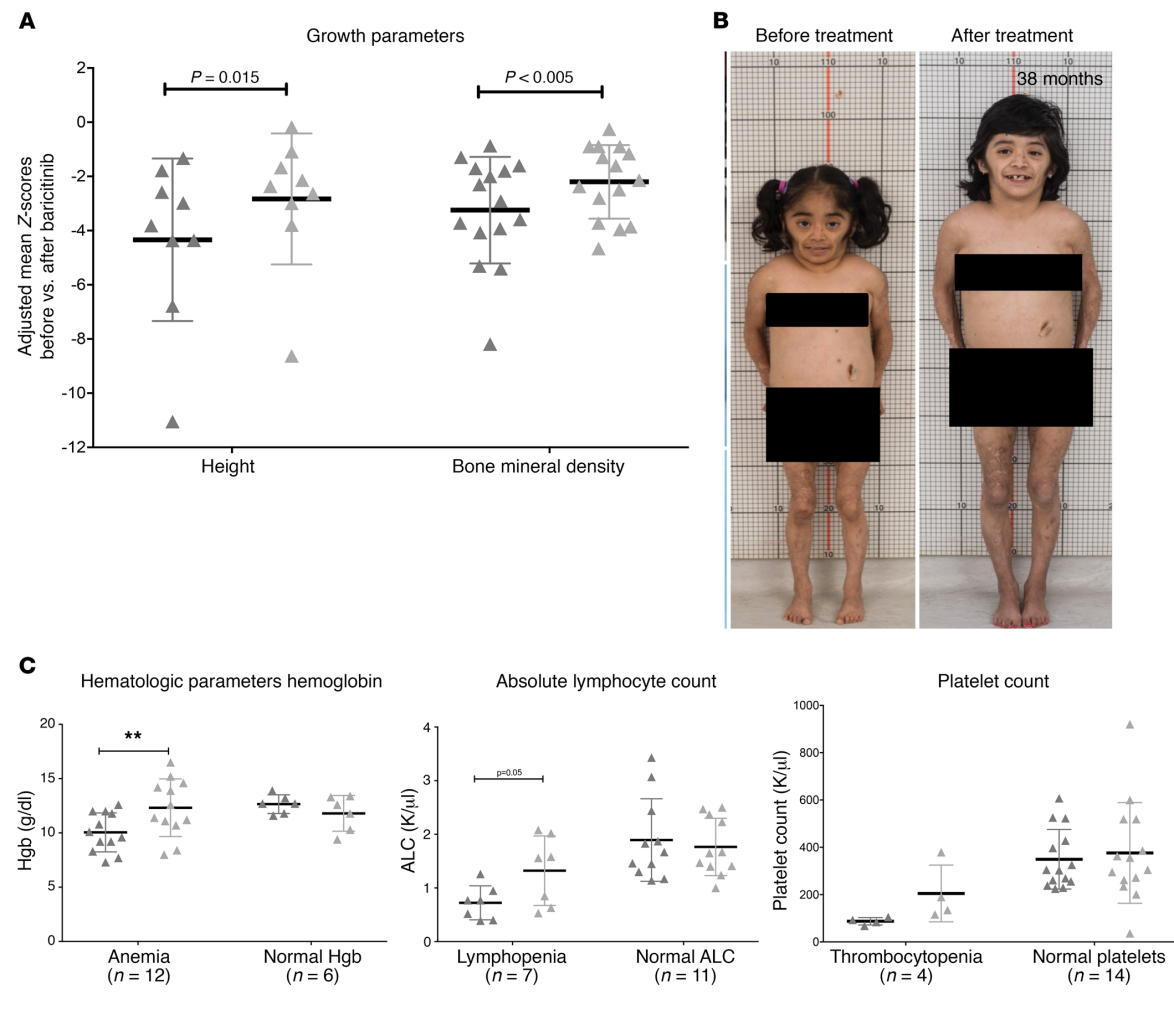
Improvement in longitudinal growth and hematologic parameters. (**A**) Clinically significant improvement was seen in height *Z*-scores and the percentiles of patients with growth potential (*n* = 13) when comparing data from before baricitinib treatment with data from the last visit. Mean height *Z*-scores improved from –4.03 ± 2.64 to –3.19 ± 2.33, with catch-up growth observed in 9 patients whose improvement translated into a mean height percentile increase from the 1.4^th^ percentile to the 7.2^nd^ percentile. (**B**) Photos of a patient with CANDLE (C8) with stunted growth since 2 years of age and a severe delay in bone age (chronological age of 14.3 years vs. bone age of 2 years). Within 30 months of treatment, her linear height increased from 90 cm to 106.8 cm, and her bone age improved from 2 years to 7.8 years. (**C**) Signs of bone marrow immunosuppression improved in all patients but 2 (C1 and O3), with increases in platelet counts, ALCs, and hemoglobin (Hgb) levels. Patient C1 continues to have persistent lymphopenia (ALC of 0.5), and patient O3 (discontinued from the program because of a poor response to treatment and osteonecrosis) had lower hemoglobin and platelet counts at the time of his last visit. This patient had multiple comorbidities including upper gastrointestinal bleeding, esophageal varices, IgA nephropathy, and idiopathic thrombocytopenia. **P < 0.05 (unadjusted) by paired Student’s *t* tests were used for both calculations; a 1-sided *t* test was used for height (in **A**) and paired Student’s *t* tests were used for all calculations, including the two asterisks (in **C**).

Prior to baricitinib treatment, the patients’ growth and physical maturation were delayed, with the mean bone age being lower by 3.49 ± 3.99 years relative to their chronological age (Supplemental Figure 3). On baricitinib, 13 patients with growth potential improved their mean height *Z*-scores from –4.03 ± 2.64 to –3.19 ± 2.33, with catch-up growth observed in 9 patients who were able to taper their corticosteroids to doses below 0.16 mg/kg/day (Figure 4, A and B, Supplemental Table 5, and Supplemental Figure 4, A–E). Bone mineral density increased, with a mean *Z*-score change from –3.25 ± 1.97 to –2.20 ± 1.36 (*P* < 0.005) (Figure 4A and Supplemental Table 6).

At baseline, 6 of 10 patients with CANDLE had metabolic syndrome; 10 patients (7 with CANDLE and 3 other patients) had hyperlipidemia; 7 pediatric patients on corticosteroids (C3, C5, C6, C7, S1, O1, and O4) met the Centers for Disease Control and Prevention (CDC) BMI criteria for obesity; and 2 patients had hepatic steatosis (1 patient with CANDLE [C5] and 1 with SAVI [S2]). On baricitinib, BMIs improved to more normal values; in 4 of 5 underweight patients (C9, C10, S4, and O3), BMI improved in 2 patients (S4 and O3) and normalized in 2 other patients (C9 and C10). For 5 of 7 obese patients, the BMI dropped to the overweight category (C3, C5, C6, S1, and O4), and 1 patient who was overweight at baseline became obese (S2) (Supplemental Table 7). The median lipid levels (HDL, LDL, and triglycerides) increased on baricitinib treatment (Supplemental Table 8). Three patients with CANDLE (C3, C4, and C8) who had hyperlipidemia at baseline developed hepatic steatosis (Supplemental Figure 5, A–C), with no improvement in the 2 patients with hepatic steatosis at baseline. In patients with CANDLE, myositis and aldolase levels improved on baricitinib treatment (*P* = 0.06) (Supplemental Table 9). In the 3 SAVI patients with baseline lung disease, signs of chronic interstitial lung disease, forced vital capacity (FVC), carbon monoxide–diffusing capacity (DLCO), and walking distance improved on baricitinib (Supplemental Table 10).

### Hematologic and immunologic markers improve on treatment with baricitinib.

At baseline, 12 of 18 (67%) patients were anemic, 7 (39%) had lymphopenia, and 4 (22%) had thrombocytopenia. The hemoglobin concentration, absolute lymphocyte count (ALC), and platelet count increased during treatment in patients with cytopenias at baseline (*P* ≤ 0.05 for hemoglobin and ALC). In patients with normal cell counts at baseline, hemoglobin and ALCs trended nonsignificantly downward (Figure 4C).

At baseline, 60% of patients with CANDLE and all patients with SAVI had detectable autoantibodies against endothelial antigens and targets including phospholipids (lupus anticoagulant and anti-cardiolipin Abs), antimyeloperoxidase and proteinase-3, and/or against nuclear antigens (ANA, SSA) and DNA (dsDNA). Autoantibody positivity significantly decreased during treatment (*P* = 0.013) (Supplemental Table 11), while cell subsets and Ig levels remained stable (Supplemental Figures 6 and 7).

### Baricitinib suppresses inflammatory markers including the IFN signature and the serum IFN cytokine IP-10.

**Figure 5:**
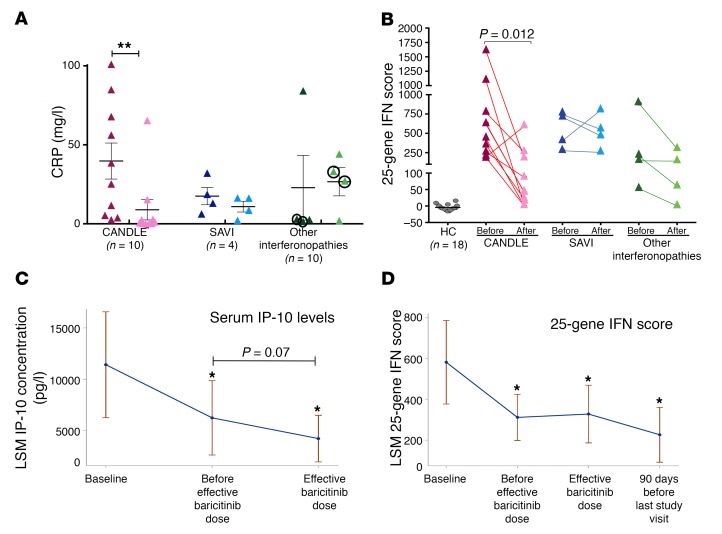
Assessment of conventional inflammatory parameters (CRP) and IFN biomarkers (serum IP-10 levels and 25-gene IFN score) with baricitinib treatment. (**A**) CRP levels dropped most significantly in CANDLE patients, with CRP levels returning to normal in 5 of 10 of these patients. The patients with other interferonopathies (O2 and O4) who stayed in the program had improved CRP levels. The 2 patients who discontinued the program because of a lack of efficacy had no improvement and are circled. **P < 0.05 (unadjusted by paired 2-sided Student’s *t*-test). Data represent the mean ± SD. (**B**) The 25-gene IFN score was graphed for the baseline score and the IFN score obtained at the last included visit only. Colors indicate data by disease, with CANDLE in red, SAVI in blue, and other interferonopathies in green. Statistical data obtained by paired 2-sided Student’s *t*-test. The IFN score normalized in 5 of 10 patients with CANDLE who achieved the remission criteria. (**C** and **D**) Longitudinally assessed serum IP-10 levels and 25-gene IFN score measurements were fitted to a repeated-measures model with “treatment phase” as a categorical independent variable. Least-squares means (LSM) of serum IP-10 and IFN response gene score with 95% CIs for each phase are graphed. *P < 0.05 (unadjusted) by paired 2-sided Student’s *t*-test.

Among the acute-phase reactants (erythrocyte sedimentation rate [ESR] and CRP), CRP levels continuously decreased with treatment, and the reduction was largest in patients with CANDLE (Table 3, Figure 5A, and Supplemental Figure 8C). The ESR did not significantly decrease and remained elevated in most patients (Supplemental Figures 8, A and B).

Biomarkers of IFN signaling, serum levels of the chemokine IP-10, and the IFN response gene score significantly decreased during treatment with baricitinib (Table 3 and Figure 5, B–D). The IFN score normalized in the 5 patients with CANDLE who achieved remission (Figure 5B). The IFN response gene score and serum IP-10 levels significantly correlated with each other (Supplemental Figure 9A). Both correlated significantly with daily symptoms (*r* = 0.26 and *r* = 0.37, *P* < 0.0001) and with lower doses of corticosteroids, indicating the ability to taper corticosteroid doses (*r* = 0.24 and *r* = 0.44, *P* < 0.005 and *P* < 0.0001, respectively) (Supplemental Table 12 and Supplemental Figures 9, B and C). The IFN biomarkers, IP-10 levels, and IFN response gene score correlated better with the ability to taper corticosteroid doses than with the acute-phase reactants (ESR and CRP). Prior to treatment, the diurnal variability of IFN scores obtained in 1 day was high. The fluctuation correlated with higher morning scores, and the daily variability was greatly reduced during baricitinib treatment when overall IFN scores decreased (Supplemental Figure 10A).

We measured IFN-α–stimulated STAT1 phosphorylation to assess type I IFN receptor responsiveness during baricitinib treatment; the IFN-α stimulation–induced STAT1 phosphorylation was reduced to the lower tertile measured in healthy controls (Supplemental Figure 10B). While patients with CANDLE were hyperresponsive to IFN-α stimulation before treatment with baricitinib (7), most patients with SAVI had maximal STAT1 phosphorylation and did not respond to IFN-α stimulation (9). On baricitinib, the IFN response in patients with SAVI recovered to the levels detected in patients with CANDLE. Other cytokines that significantly decreased in baricitinib-treated patients included MCP-1, granulocyte-macropage CSF (GM-CSF), IL-15, and IL-5 (Supplemental Figure 11).

### Safety summary.

Overall, baricitinib was well tolerated. At the time of safety analysis (June 2017), the mean baricitinib exposure was 3.5 years (range, 2.3–5.6 years for ongoing patients), representing 63 patient years of exposure. No deaths were reported during the program. Two patients (11%) with inadequate responses discontinued treatment because of adverse events. One patient (O3) with an undifferentiated interferonopathy had evidence of osteonecrosis (right femur) 3 days after starting baricitinib and was taken off the drug because of progression after 14 weeks of baricitinib treatment. Eighteen months after discontinuing baricitinib, the patient died as a result of a worsening of preexisting nodular regenerative hyperplasia and portal hypertension complicated by recurrent esophageal variceal hemorrhages, IgA nephropathy, and renal insufficiency. One patient with CANDLE (C7) developed azotemia in the context of BK viruria and viremia and discontinued treatment after 117 weeks because of acute kidney injury. This patient died 4 months later as a result of exacerbation of CANDLE syndrome, in the context of a respiratory tract infection and interstitial lung disease. Fifteen patients (83%) had at least 1 serious adverse event (SAE) (Supplemental Table 13). In most instances, the SAEs resolved without interruption of baricitinib treatment. Treatment-emergent infections were observed in 16 patients (89%) (Table 4). Upper respiratory tract infections were most frequent. Two patients developed herpes zoster, with unilateral lesions restricted to 2 to 3 contiguous dermatomes. Transient cytopenias developed in the context of infections and intermittent disease exacerbations (Supplemental Tables 14 and 15). An unexpected finding was the development of polyomavirus (BK) viremia in patient C7. While 2 patients had low-titer intermittent BK viremia before baricitinib treatment, 8 additional patients developed intermittent BK viremia during baricitinib treatment. In contrast to the first patient who had high-titer BK viremia in the context of worsening renal disease, the copy number in the other patients was low and variable, with stable low-copy-number viremia and stable renal function over time (Supplemental Table 16).

## Discussion

We found that treatment with baricitinib improved disease signs and symptoms and allowed a significant reduction of corticosteroid treatment in patients with CANDLE and SAVI and in 2 patients with other interferonopathies in an expanded access program. Of the 10 patients with CANDLE, 5 (50%) patients were able to permanently discontinue corticosteroid therapy, without a return of disease symptoms; their inflammatory markers normalized, and they achieved durable inflammatory remission on baricitinib. In patients with SAVI, baricitinib treatment improved the vasculitis flares and prevented the progression of spontaneous amputations and the development of gangrene. Baricitinib also stabilized interstitial lung disease by preserving pulmonary function indices including DLCO and improved walking distances. Despite these clinical improvements, inflammatory markers did not normalize in any of the patients with SAVI, and although IFN scores decreased, the absolute levels remained elevated. These findings are consistent with 2 previous reports on a total of 7 patients with SAVI who were treated for 3 to 15 months with the JAK inhibitor ruxolitinib (17, 18).

Among the 4 patients with other (initially uncharacterized) interferonopathies, 2 patients, for whom a genetic diagnosis could not be established did not respond and discontinued treatment. The 2 responders had both severe panniculitis and lipoatrophy. One of the patients had peripheral vasculitis, and at the age of seven years developed moyamoya-like cerebral vasculopathy resulting in vascular occlusion and stroke. She was later found to be homozygous for a *SAMHD1* deletion, which allowed a retrospective diagnosis of later-onset AGS5 (19). The second patient had nodular panniculitis, lipoatrophy, and marked systemic inflammation. This patient was later found to have a novel frameshift mutation in *SAMD9L,* suggesting a possible novel interferonopathy. The 2 responders had higher 25-gene IFN scores at baseline compared with those of the 2 nonresponders, thus suggesting that a combination of clinical phenotype and grossly elevated IFN scores might be useful in predicting responses to IFN-blocking treatments such as JAK1/2 inhibition.

Most patients with CANDLE and SAVI and some other interferonopathies have significant growth and bone maturation delays and low bone mineral density. The significant improvement in height and bone mineral density *Z*-scores demonstrates that the baricitinib dosing regimen we developed to optimize disease control allows for catch-up growth and bone production, which was most prominent in patients who were able to reduce corticosteroid doses to less than 0.16 mg/kg/day. These observations ease concerns that JAK inhibitors could reduce growth hormone (GH) function through inhibition of GH receptor–induced tyrosine kinase JAK2 phosphorylation (20, 21). Despite improvement in inflammatory markers, hyperlipidemia and hepatic steatosis did not improve in the patients with CANDLE. In fact, 3 patients with CANDLE on baricitinib treatment developed hepatic steatosis, which manifested as a moderate decrease in their BMIs from baseline, pointing to a role of proteasome dysfunction in the development of hepatic steatosis that is independent of IFN-mediated inflammation (22).

The abnormal IFN responses in vivo were modulated by reducing IFN-α–induced STAT1 phosphorylation and downstream IFN targets such as serum IP-10 levels and an IFN response gene score after treatment with baricitinib. The reduction in IFN biomarkers correlated with the improvement of clinical signs and symptoms and with the ability to taper the corticosteroid dose. Peripheral blood mononuclear cells (PBMCs) from most untreated patients with SAVI showed high constitutive STAT1 phosphorylation and were unresponsive to further IFN-α stimulation (9). However, we observed decreased constitutive activation and restored type I IFN receptor responsiveness with baricitinib treatment. We also observed reductions in serum levels of GM-CSF and its downstream mediator, the chemokine MCP-1 (CCL2), with baricitinib treatment (23). GM-CSF promotes myeloid differentiation toward inflammatory, M1-like macrophages, and CCL2 promotes monocyte/macrophage recruitment to sites of tissue injury or infection (24–26). GM-CSF, particularly in the context of IFNs, primes monocytes to amplified stimulation-induced cytokine and chemokine overproduction that includes the production of IFN biomarkers (27) through epigenetic remodeling (28). The reduction in diurnal variability of the IFN response gene score and the modulation of IFN-α–induced STAT1 phosphorylation in baricitinib-treated patients may be consistent with a reset of the IFN receptor sensitivity through epigenetic remodeling mediated by JAK inhibition, a mechanism that was recently suggested in a murine model treated with IFN-α and a JAK inhibitor (29). The impact of JAK inhibition on epigenetic remodeling as a molecular mechanism that may attenuate an IFN-dependent amplification loop (1) needs further evaluation.

Overall, the mean drug exposure levels to optimize disease control in the context of an acceptable safety profile in our patients were 1.83-fold higher than for patients with rheumatoid arthritis taking 4 mg/day baricitinib (30); the severe disease manifestations and the need to target type I IFN signaling are likely reasons for the higher exposures in our patients.

Treatment with baricitinib was overall well tolerated. Consistent with studies of adult patients with rheumatoid arthritis, upper respiratory tract infections were the most frequently reported treatment-emergent adverse events, and 2 patients developed herpes zoster (30, 31). However, the observation of viral reactivation with BK virus (BK viremia and viruria) was unique in this patient population. Patients’ viral titers in the urine and blood remained stable over a 2-year period on treatment. While the clinical significance of measurable BK titers remains uncertain (32–34), viral titers in the blood should be monitored in this vulnerable population.

Although the number of patients enrolled was small, the long duration of treatment (2.3–5.6 years and continuing), the durable responses to treatment, and the reduction in inflammatory markers confirm the long-lasting effect of baricitinib treatment.

In summary, our data show that clinical signs and symptoms of patients with CANDLE and SAVI improved with baricitinib treatment. The decrease in systemic and organ-specific inflammation in terms of IFN biomarker reduction indicates a causative role for chronic IFN signaling in disease pathogenesis in patients with type I IFN–mediated diseases (1, 35).

## Methods

### Patients

Patients with genetically confirmed CANDLE or SAVI or a suspected undifferentiated interferonopathy, who were referred to our center for evaluation and treatment recommendations and who were 17.5 months of age or older, weighed 8.5 kg or more, and had active clinical disease (CANDLE diary score ≥0.5 or a SAVI diary score ≥1.0) were eligible to participate in this study. Patients had to receive or to have previously failed treatment with oral corticosteroids (≥0.15 mg/kg/day prednisone or its equivalent).

### Program design and treatment

The open-label expanded access program (ClinicalTrials.gov NCT01724580) provides access to baricitinib to eligible patients who are without other satisfactory treatment options. Data presented here are from patients enrolled at the NIH. Data were collected in a company-provided database and in a clinical database at the NIH.

#### Treatment with the JAK inhibitor baricitinib and dosing adjustments and escalation.

At the start of the program, pediatric pharmacokinetic (PK) data were not available, and baricitinib was started orally at 100 μg once a day. As PK and clinical response data became available, the dose escalation scheme for baricitinib was modified (36). An inadequate response, defined as elevated average diary scores and active clinical disease (diary scores ≥0.5 for CANDLE or ≥1 for SAVI, or ongoing clinical symptoms of disease activity), in the absence of signs of drug toxicity (i.e., drop in hemoglobin), allowed increases in the daily dose of baricitinib. Dose escalations were initially allowed when the PK baricitinib peak and trough levels were within the range of those observed in healthy adult control subjects or in patients with RA or psoriasis (Eli Lilly internal data); further dose increases were approved in amendments and monitored according to the PK data. We determined the visit at which the enrolled patients reached optimal tolerated dosing for baricitinib (Supplemental Table 1). Population pharmacokinetic (PopPK) analyses were performed at optimized tolerated baricitinib doses (36). The mean exposures, measured as AUC_24,SS_, were 1.83-fold higher than those obtained in adult RA patients receiving oral baricitinib doses at 4 mg once daily in phase III studies. A dosing table has been published previously (36).

### Clinical benefit assessment

We collected limited efficacy data on the expanded access protocol (diary scores, corticosteroid doses, medications, safety data) to aid in the determination of dose titration and ensure evidence of the benefit justifying the risk. Additional efficacy data were collected according to the natural history protocol or as part of routine patient care (see Supplemental Methods).

#### DDS score.

Primary benefit was defined as a decrease in the DDS score to less than 0.5 for patients with CANDLE and other interferonopathies and to less than 1.0 for patients with SAVI. The cutoffs corresponded with clinically meaningful responses to treatment. Each patient or caregiver was instructed to complete the diary at approximately the same time every day and to rate the impact of each symptom on the patient. The average score for each symptom was calculated and summed and divided by the number of assessed symptoms (see Supplemental Methods). Diary scores were used in the assessment of disease activity, the need for baricitinib dose increases, and the initiation of steroid dose tapering. Diary scores were collected for 2 weeks prior to baricitinib administration and every day throughout the program. Patient O3 completed diaries for 76.7% of the days, and all of the other patients completed diaries for more than 92% of the days (Supplemental Table 2).

#### Reduction in daily corticosteroids.

Secondary benefit was assessed for patients on treatment with corticosteroids at enrollment. Successful reduction was defined as a reduction in the corticosteroid dose to less than 0.15 mg/kg/day of the prednisone equivalent or a decrease of at least 50% of the patient’s daily dose at baseline.

#### Other clinical outcome measures.

We measured clinical remission, change in disability, quality of life, and patient and physician global assessments. *Z*-scores for height, weight, BMI, bone age, and bone mineral density were calculated at baseline and at respective follow-up visits (see Supplemental Methods). CANDLE-specific outcomes included an assessment of hyperlipidemia and hepatic steatosis (37). SAVI-specific outcomes included an assessment of interstitial lung disease by PFTs, a 6-minute walking test (6MWT), and a yearly chest CT (see Supplemental Methods).

### Immunological evaluation

The immunological evaluations included the measurement of inflammatory markers, high-sensitivity CRP (hsCRP) and ESR, the autoantibody numbers, lymphocyte subset panels, Ig levels, and hematologic values. IFN signaling was assessed by a STAT1 phosphorylation assay, quantification of a 25-gene IFN gene score (referred to as IFN response gene score) in whole blood (12), and measurement of serum IP-10 and other cytokine levels (see Supplemental Methods).

### Safety assessment

The development of comorbidities and hospitalizations was documented. The NIH’s Common Terminology Criteria for Adverse Events (CTCAE), version 4.03, was used to categorize abnormal results after enrollment. Vital signs including weight and height, clinical laboratory tests, a complete blood cell count with differential, renal and liver function, lipid profile, urinalysis, and other safety assessments were performed at all protocol visits. BK titers were measured after the discovery of the first BK virus case among the study participants in June 2015 (Supplemental Methods).

### Statistics

#### Post hoc analyses to assess relationships between treatment and clinical outcomes, conventional biomarkers (ESR and CRP), and IFN biomarkers.

As this was an expanded access protocol and the medical conditions treated in this program are rare, we anticipated that the number of enrollees would be small, and therefore no formal statistical analyses were planned. Null and alternative hypotheses were not defined prospectively, and analyses were not adjusted for multiple comparisons. We assessed the mean change in clinical measures obtained under the expanded access program, including diary scores, prednisone doses, and data collected under the natural history study by comparing data at baseline (before treatment) and at the last NIH visit. A 2-sided Student’s *t* test was used for parametric data and the Wilcoxon signed-rank test for nonparametric data, at a *P* value of less than 0.05. The cutoff date for expanded access program data was October 31, 2016, for natural history outcomes, February 22, 2017, and for safety data, June 5, 2017.

#### Assessment of dose-response relationships of clinical and biomarker measures.

As the dose of baricitinib was titrated to the clinical response, we determined the visit at which the patients were started on the optimal tolerated baricitinib doses (Supplemental Table 1). For each patient, the timeline was divided into 4 study phases, and the primary outcome was averaged over each period, resulting in 4 averages per period per patient. Phase 1 (baseline) included the 29 days prior to the baricitinib start date and the first day of treatment; phase 2 (post-treatment preoptimal baricitinib dose period) included the dose escalation period, which started with the second day of treatment and continued up to the first day that an optimal tolerated dose was achieved; phase 3 (post-optimal tolerated dose period) started with the day the optimal tolerated dose was achieved and continued until 90 days before the end of the study evaluation; and phase 4 included the last 90 days before the last included visit (all patients were on optimal tolerated doses) (Supplemental Table 1). To confirm trends in longitudinally collected data (diary scores, prednisone dose, acute phase reactants and IFN biomarkers), the data were fitted to a repeated-measures model with “phase” as a categorical independent variable. The least-squares means with 95% CIs for each phase were assessed. The Cochran-Mantel-Haenszel test was used to analyze the association between binary outcomes.

#### Assessment of associations between clinical outcomes, IFN biomarkers, and conventional biomarkers (ESR and CRP).

A linear mixed model with a random slope and intercept and an unstructured variance-covariance matrix was used to assess the association of clinical outcome measures (DDS and corticosteroid dose) with biomarkers of IFN signaling (serum IP-10 levels and the 25-gene IFN score. To determine the correlation between repeated measures, we applied a method based on a reconfiguration of the X and Y variables by using the SAS PROC MIXED statement (38).

### Study approval

The program was approved by the IRBs of the NIH, NIAMS, NIDDK, and NIAID. All patients were coenrolled in the NIH Natural History Protocol of Autoinflammatory Diseases (NCT02974595), under which clinical and biomarker data were assessed. Patients or their parents provided written informed consent. Additional written photo consent was obtained from patients included in this manuscript. Patients were evaluated at baseline, monthly for the first 12 months, and every 3 months thereafter. The program is currently ongoing.

### Authors contributions

GAMS acquired and analyzed data, oversaw the clinical and regulatory aspects of the study, and participated in writing the manuscript. AR, SR, HW, PJH, YB, SS, SM, JAD, DB, DLS, LG, TK, DF, DCC, HK, SD, RAC, LF, BK, MOB, SMP, A. Brofferio, AS, EWC, JGD, CH, SMH, JF, ASA, SO, and PAB acquired and interpreted clinical data. AADJ, A. Biancotto, and MG conducted experiments and acquired and analyzed data. JCR, LRF, KRC, NW, AJL, RJB, KIR, TH, KMB, PK, SRB, MW, HKS, and VN acquired and analyzed clinical and biomarker subspecialty data. MS, AP, and JMJ summarized and analyzed the safety data and participated in writing the manuscript. PGW conducted and oversaw the statistical analyses of the study data. WLM and RGM designed the expanded use program, reviewed and analyzed the data, and wrote the manuscript. GAMS and RGM wrote the first draft of the manuscript. All authors reviewed and approved the final version of the manuscript.

## Supplementary Material

Supplemental data

## Figures and Tables

**Table 4:**
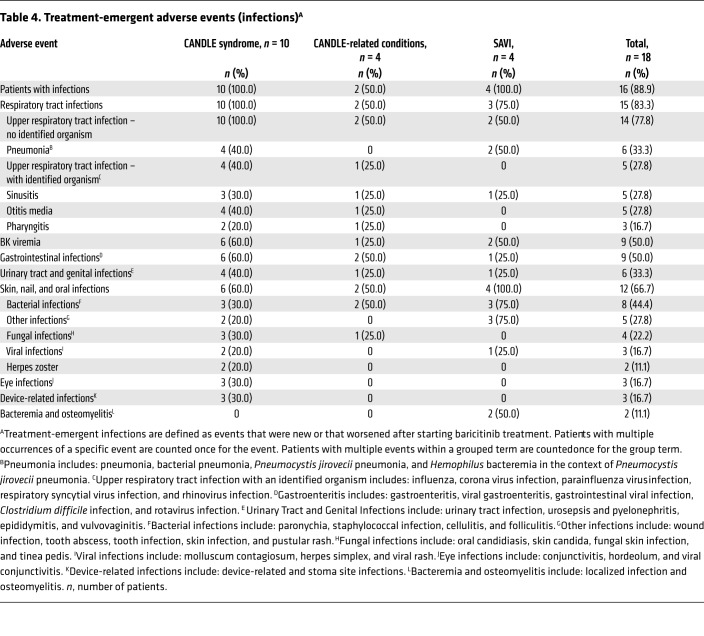
Treatment-emergent adverse events (infections)^A^

**Table 3:**
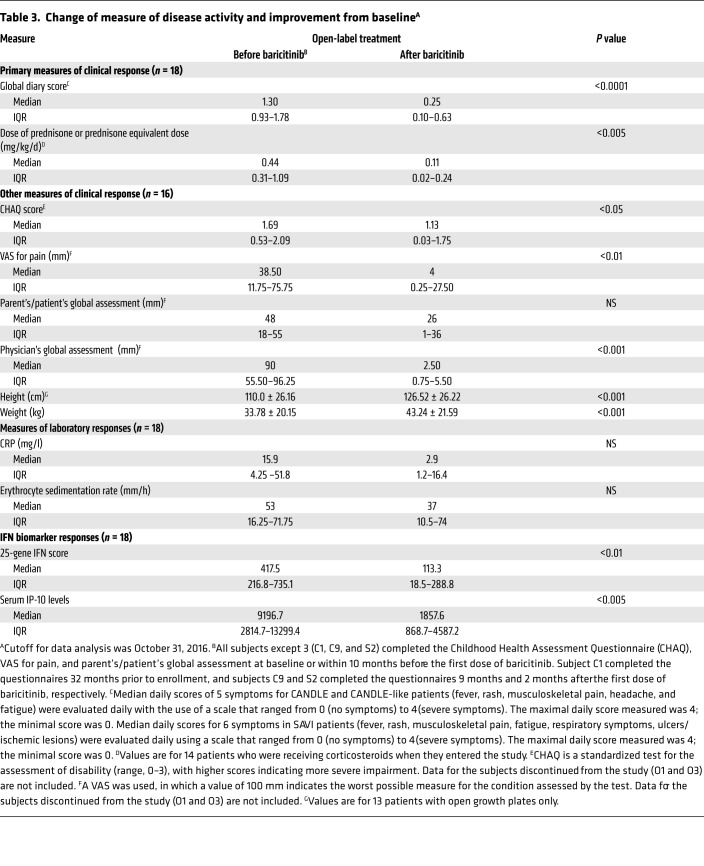
Change of measure of disease activity and improvement from baseline^A^

**Table 2:**
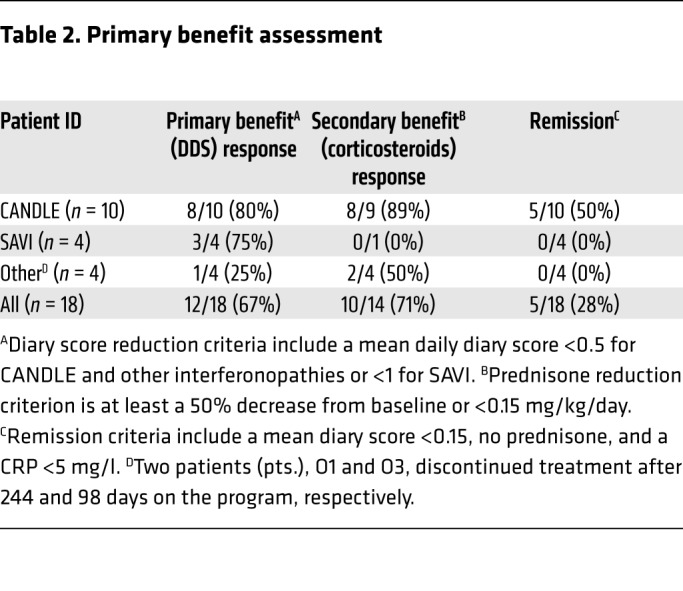
Primary benefit assessment

**Table 1:**
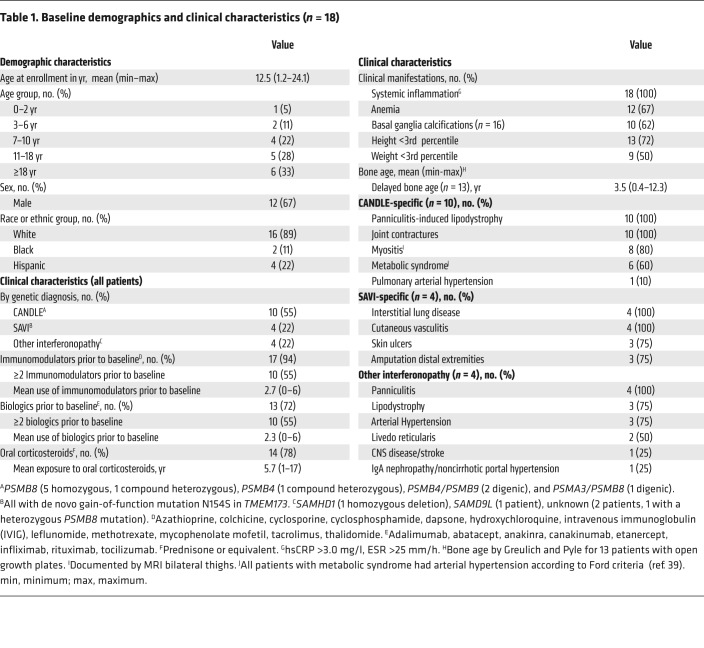
Baseline demographics and clinical characteristics (*n* = 18)

## Version 1 04/12/2018

In-Press Preview

## Version 2 06/11/2018

Electronic publication

## Version 3 07/02/2018

Print issue publication
