# Supplementary material for: JAK1/2 inhibition with baricitinib in the treatment of autoinflammatory interferonopathies
Source: J Clin Invest. 2018 Jun 11;128(7):3041–52. doi: 10.1172/JCI98814 (PMC6026004; doi:10.1172/JCI98814)
Supplement: Supplemental data [file jci-128-98814-s313.pdf]

Supplemental Data

JAK 1/2 inhibition with baricitinib in the treatment of autoinflammatory interferonopathies

Gina A. Montealegre Sanchez, M.D., MS[1], Adam Reinhardt, M.D[2], Suzanne Ramsey, M.D[3], Helmut Wittkowski, M.D[4], Philip J. Hashkes, M.D[5], Yackov Berkun, M.D[6], Susanne Schalm, M.D[7], Sara Murias, M.D[8], JA Dare, M.D[9], Diane Brown, M.D., Ph.D [10], Deborah L. Stone, M.D[11], Ling Gao, M.D[9], Thomas Klausmeier, M.D[12], Dirk Foell, M.D[4], Adriana A. de Jesus M.D., PhD[1], Dawn C. Chapelle, CRNP[13], Hanna Kim, M.D.,MS[13], Samantha Dill, CRNP[13], Robert A. Colbert, M.D., Ph.D[13], Laura Failla, FNPC[1], Bahar Kost[13], Michelle O'Brien, RN[13], James C. Reynolds, M.D[14], Les R. Folio, DO., MPH[14], Katherine R. Calvo, M.D., Ph.D[14], Scott M. Paul, M.D[14] , Nargues Weir, M.D[15], Alessandra Brofferio, M.D[15], Ariane Soldatos, M.D[16], Angelique Biancotto, Ph.D[15], Edward W. Cowen, M.D[13], John G. Digiovanna, M.D[17], Massimo Gadina, Ph.D.[13], Andrew J. Lipton, M.D., MPH&TM[18], Colleen Hadigan, M.D[19], Steven M. Holland, M.D[19], Joseph Fontana, M.D[15], Ahmad S. Alawad, M.D[20],Rebecca J. Brown, M.D[20], Kristina I. Rother, M.D[20], Theo Heller, M.D[20], Kristina M. Brooks, PharmD[14], Parag Kumar, PharmD[14], Stephen R. Brooks, Ph.D[13], Meryl Waldman, M.D[20], Harsharan K. Singh, M.D[21], Volker Nickeleit, M.D[21], Maria Silk, PharmD[22], Apurva Prakash[22], Jonathan M. Janes, FRCP[22], Seza Ozen, M.D[23], Paul G. Wakim, PhD[24], Paul A. Brogan, M.D[25], William L. Macias, M.D., PhD[22] and Raphaela Goldbach-Mansky, M.D., MHS[1]

[1]TADS/NIAID/NIH, Bethesda, Maryland, United States; [2]Faculty of Physicians of the University of Nebraska Medical Center, College of Medicine, Omaha, Nebraska, United States; [3]IWK Health Centre, Halifax, Canada; [4] Department of Pediatric Rheumatology and Immunology, University Children's Hospital, Muenster, Germany; [5]Shaare-Zedek Medical Center, Jerusalem, Israel; [6]Hadassah Hebrew University Medical Center, Jerusalem, Israel; [7]Hauner Children's Hospital LMU, Munich, Germany; [8]Hospital Infantil La Paz, Madrid, Spain; [9]University of Arkansas for Medical Sciences, Little Rock, Arkansas, United States; [10]Children's Hospital Los Angeles, California, United States; [11]NHGRI/NIH, Bethesda, Maryland, United States;[12]Riley Hospital for Children, Indianapolis, Indiana, United States;[13]NIAMS/NIH, Bethesda, Maryland, United States; [14]CC/NIH, Bethesda, Maryland, United States; [15]NHLBI/NIH, Bethesda, Maryland, United States;[16]NINDS/NIH, Bethesda, Maryland, United States; [17]NCI/NIH, Bethesda, Maryland, United States; [18]WRNMMC, Bethesda, Maryland, United States; [19]NIAID/NIH, Bethesda, Maryland, United States; [20]NIDDK/NIH, Bethesda, Maryland, United States; [21]University of North Carolina School of Medicine, Chapel Hill, North Carolina, United States; [22]Eli Lilly and Company, Indianapolis, Indiana, United States; [23]Hacettepe University Faculty of Medicine, Ankara, Turkey;[24]Biostatistics and Clinical Epidemiology Service, NIH Clinical Center, Bethesda, Maryland, United States; [25]UCL Great Ormond Street Institute of Child Health and Great Ormond Street Hospital NHS Foundation, London, UK

This research was supported by the Intramural Research Program of the NIH, NIAID and NIAMS. The views expressed in this article are those of the authors and do not reflect the official policy of the Department of Army/Navy/Air Force, Department of Defense or U.S. Government.

CONTENTS

I. Supplemental Methods

A. PATIENTS AND INCLUSION AND EXCLUSION CRITERIA
B. CLINICAL BENEFIT ASSESSMENT
C. DISEASE-SPECIFIC OUTCOMES
D. IMMUNOLOGICAL ASSESSMENTS
E. SAFETY ASSESSMENT
F. STATISTICAL ANALYSES

II. Supplemental Tables

1. **Supplemental Table 1. Baricitinib initial dosing, time to reach optimal tolerated doses**
2. **Supplemental Table 2. Percent completion of diary data for all patients**
3. **Supplemental Table 3: Summary of primary outcomes (diary score and steroid dose), and on achieving clinical remission**
4. **Supplemental Table 4. Stable and continued remission in 5 patients with CANDLE who achieved remission criteria on baricitinib treatment**
5. **Supplemental Table 5. Changes in height Z-scores on baricitinib**
6. **Supplemental Table 6. Changes in Dual-energy x-ray absorptiometry (DEXA) Z-scores on baricitinib treatment**
7. **Supplemental Table 7. Changes in Body Mass Index (BMI) on baricitinib**
8. **Supplemental Table 8. Changes in lipid profile and lipid ratios on baricitinib treatment**
9. **Supplemental Table 9. Changes in muscle enzymes in CANDLE patients**
10. **Supplemental Table 10. Changes in pulmonary function test and chest CT in SAVI patients**
11. **Supplemental Table 11. Change of antibody profile in CANDLE, SAVI and other IFNopathies on baricitinib**
12. **Supplemental Table 12. Association of IFN biomarkers (IP-10 and 25-gene IFN score) with Clinical Outcomes and with historical biomarkers (ESR and CRP)**

13. **Supplemental Table 13. Serious Adverse Events (SAE) per patient**

14. **Supplemental Table 14. Treatment-emergent Categorical Laboratory Results of Interest**

15. **Supplemental Table 15. Treatment-emergent Adverse Events (non-infection) by System/Organ Group with Individual Event Terms Shown if in ≥4 Patients**

16. **Supplemental Table 16. BK virus titers initial vs. last included assessment**

III. Supplemental Figures

1. **Supplemental Figure 1. Patient enrollment and disposition**

2. **Supplemental Figure 2. Improvement in clinical disease manifestations in patients with CANDLE and other IFNopathies treated with baricitinib**

3. **Supplemental Figure 3. Delay in bone age at baseline**

4. **Supplemental Figures 4A-E. Anthropometric measurements in patients with CANDLE without growth potential (C4, C9 and C10), and with growth potential (C2, C3, C5, and C1, C6, C7 and C8), in patients with SAVI and with other IFNopathies**

5. **Supplemental Figures 5A-C. Metabolic syndrome in patients with CANDLE vs. SAVI**

6. **Supplemental Figure 6. Cell subsets in CANDLE, SAVI and other IFNopathies**

7. **Supplemental Figure 7. Quantitative immunoglobulins in CANDLE, SAVI and other IFNopathies**

8. **Supplemental Figure 8. Erythrocyte sedimentation rate (ESR) and C-reactive protein (CRP) response to baricitinib**

9. **Supplemental Figure 9. Evaluation of biomarkers of IFN signaling and correlation with daily symptoms and ability to wean corticosteroids**

10. **Supplemental Figure 10A,B. Evaluation of biomarkers of IFN signaling**

11. **Supplemental Figure 11. Cytokine changes on baricitinib**

I. Supplemental Methods

A. PATIENTS AND INCLUSION AND EXCLUSION CRITERIA

Patients who met all of the inclusion criteria and did not meet any of the exclusion criteria were considered for the program. All patients were required to meet the enrollment criteria to be eligible to receive baricitinib. The program was conducted in accordance with the ethical principles of the Declaration of Helsinki and Good Clinical Practice guidelines.

Inclusion Criteria:

1. Have systemic signs and symptoms of inflammation as manifested by the presence of 2 or more of the following symptoms: rash, fever, musculoskeletal pain, headache, fatigue, weakness, respiratory/breathing symptoms, or ulcers/ischemic lesions.
2. Have an average daily diary score of ≥0.5 (CANDLE diary) or ≥1.0 (SAVI diary) assessed over at least 2 consecutive weeks during the 6 weeks prior to entry, if available. Otherwise, patients can complete the diary after consent is signed during the screening period and meet the inclusion criteria for enrollment into the program.
3. Are ≥17.5 months of age.
4. Are ≥8.5 kg in body weight.
5. Have been previously treated with at least 1 biologic therapy and, in the opinion of the investigator, did not respond or are no longer responding to therapy. If the patient has been diagnosed with CANDLE or an equivalent syndrome with decreased proteasome function, or SAVI, the need for previous biologic therapy is not required.
6. Require treatment with oral corticosteroids (≥0.15 mg/kg/d of prednisone or its equivalent) for control of systemic signs and symptoms of their chronic inflammatory disease for at least 2 weeks prior to entry, or in the opinion of the investigator, have failed an adequate course of steroids.
7. Have had previous documented elevations in acute-phase reactants (for example, high sensitivity C-reactive protein) considered to be the result of the inflammatory disease (patients with CANDLE or CANDLE-related conditions only).
8. Have the ability to provide informed consent or have a legal representative who is willing and able to provide written informed consent, provided that assent is obtained from patients at an age-appropriate level.

Inclusion Criteria for patients with CANDLE-Related Conditions

Patients with CANDLE-related conditions were eligible for entry into the program, only if they met all of the common inclusion criteria (1 through 8) and all of the following criteria:

9. Have organ specific inflammation involving at least one of the following: vasculopathy (such as arterial hypertension, pulmonary hypertension, vascular calcifications such as basal ganglia calcifications, or livedo reticularis), metabolic changes (such as lipodystrophy, dyslipidemia, insulin resistance, or intra-abdominal fat deposition), musculoskeletal manifestations (myositis, arthralgias or arthritis, and/or panniculitis), hematological manifestations (i.e. cytopenias) and/or interstitial lung disease.
10. Have a history of high IP-10/CXCL10 levels and/or IFN response signature in peripheral blood mononuclear cells being one of the most dysregulated blood signatures.

Exclusion Criteria

Patients were excluded from the program if they met any of the following criteria:

1. Have received an immunosuppressive biologic agent/monoclonal antibody within 4 half-lives prior to entry. Use of Intravenous Immunoglobulin (IVIG) is permitted.
2. Are pregnant or nursing at the time of entry.
3. Are females of childbearing potential who do not agree to use 2 forms of highly effective birth control when engaging in sexual intercourse with a male partner while enrolled in the program and for at least 4 weeks following the last dose of investigational product.
4. Are males who do not agree to use 2 forms of highly effective birth control while engaging in sexual intercourse with female partners of
   childbearing potential while enrolled in the program and for at least 4 weeks following

the last dose of investigational product.

5. Have had symptomatic herpes zoster infection within 12 weeks prior to entry or during the screening period.
6. Have a history of disseminated/complicated herpes zoster.
7. Have evidence of active infection, at the time of entry or during the screening period, that, in the opinion of the investigator, would pose an unacceptable risk for participating in the program.
8. Have a history of active hepatitis B, hepatitis C, or human immunodeficiency virus (HIV).
9. Have documented high titer autoantibodies suggestive clinically of autoimmune diseases other than severe JDM.
10. Are immunocompromised and, in the opinion of the investigator, are at an unacceptable risk for participating in the program.
11. Have had a serious systemic or local infection within 12 weeks prior to entry or during the screening period. Exceptions include SAVI patients with infected ulcerative skin lesions, which in the opinion of the investigator would not pose an unacceptable risk for participating in the program.
12. Have been exposed to a live vaccine within 12 weeks prior to entry or are expected to need/receive a live vaccine during the course of the program.
13. Have had household contact with a person with active tuberculosis (TB) and did not receive appropriate and documented prophylaxis for TB.
14. Have a serious and/or unstable illness that, in the opinion of the investigator, poses an unacceptable risk for the patient's participation in the program.
15. Have an estimated glomerular filtration rate (eGFR) based on the most recent available serum creatinine of <40 mL/min/1.73 m2.
16. Have or have had a history of lymphoproliferative disease; or signs or symptoms suggestive of possible lymphoproliferative disease, or active primary or recurrent malignant disease; or been in remission from clinically significant malignancy for <5 years.
17. Have a history of chronic alcohol abuse or intravenous drug abuse within the 2 years prior to entry.
18. Are unable or unwilling to make themselves available for the duration of the program and/or are unwilling to follow protocol restrictions/procedures.
19. Are investigator site personnel directly affiliated with this program and/or their immediate families. Immediate family is defined as a spouse, parent, child, or sibling, whether biological or legally adopted.
20. Are currently enrolled in, or discontinued within the last 30 days from, a clinical trial involving an investigational product or non-approved use of a drug or device (other than the investigational product used in this program), or concurrently enrolled in any other type of medical research judged not to be scientifically or medically compatible with this program.

Entered patients were ineligible for enrollment (that is, ineligible to receive baricitinib) and were discontinued from the program if they met any of the following criteria:

21. Have screening laboratory test values outside the reference range for the population or investigative site that, in the opinion of the investigator, pose an unacceptable risk for the patient's participation in the program.
22. Have any of the following specific abnormalities on screening laboratory tests:
    - AST or ALT >2 × ULN unless the hepatitis is confirmed as resulting from the autoinflammatory condition. Even if inflammatory myositis is considered present, AST or ALT cannot exceed 5 × upper limit of normal (ULN).
    - Hemoglobin <10 g/dL (100 g/L). Patients may be enrolled with hemoglobin <10 g/dL if the anemia is considered a result of the underlying disease (see below).
    - Total WBC count <2500 cells/μL. Patients may be enrolled with WBC count <2500 cells/μL if the low WBC count is considered a result of the underlying

disease (see below).

- Neutropenia (absolute neutrophil count [ANC] <1200 cells/µL). Patients may be enrolled with an ANC <1200 cells/µL if the low ANC is considered a result of the underlying disease (see below).
- Thrombocytopenia (platelets <100,000/µL). Patients may be enrolled with a platelet count <100,000/µL if the low platelet count is considered a result of the underlying disease (see below).
- eGFR <40 mL/min/1.73 m2

Note: A patient with CANDLE, CANDLE-related condition, or SAVI may be enrolled with any of the above specific abnormalities on screening laboratory tests if these laboratory abnormalities are considered a feature of the disease. An expert independent of the principal investigator (preferably a hematologist) must evaluate the patient and, in conjunction with the principal investigator, document that the laboratory abnormality is a feature of the underlying CANDLE, CANDLE-related condition, or SAVI condition; the investigator must also consult with the Sponsor before the patient can be enrolled.

23. Have screening thyroid-stimulating hormone and/or thyroxine values outside of the laboratory's reference range and are assessed to be clinically significant. Patients who are receiving thyroxine as replacement therapy may participate in the program, provided stable therapy has been administered for ≥3 months and thyroid-stimulating hormone is within the laboratory's reference range.
24. Have screening electrocardiogram (ECG) abnormalities that, in the opinion of the investigator, are clinically significant and indicate an unacceptable risk for the patient's participation in the program (for example, Bazett's corrected QT interval >450 msec for males and >470 msec for females).
25. Have evidence of active or latent TB as documented by a positive purified protein derivative (PPD) test. Exceptions include
patients with a history of latent TB who have documented evidence of completing a course of appropriate treatment:
26. Have a positive test for hepatitis B defined as (1) positive for hepatitis B surface antigen, or (2) positive for anti-hepatitis B core antibody, but negative for hepatitis B surface antibody unless the anti-hepatitis B core antibody is thought to be a false positive result. In the latter case, confirmation of the presence of hepatitis B virus (HBV) by DNA testing is required. An HBV DNA indeterminate result is considered HBV infection.
27. Have hepatitis C virus (positive for anti-hepatitis C antibody with confirmed presence of hepatitis C virus); have evidence of HIV infection, and/or positive HIV antibodies.

B. CLINICAL BENEFIT ASSESMENT

The outcomes and measurements marked with an asterisk (*) were collected under the natural history protocol or are part of routine care of patient with chronic inflammation who are or are not receiving corticosteroids.

Daily diary score (DDS) assessment: Disease-specific patient diaries (for CANDLE and SAVI patients) were provided for daily collection of information on patients' signs and symptoms. CANDLE and other interferonopathy patients or their parents recorded daily symptoms of fever, rash, musculoskeletal pain, headaches and fatigue; and SAVI patients recorded, fever, rash, musculoskeletal pain, fatigue, respiratory symptoms and severity of ulcers/ischemic lesions. Each symptom was rated on a scale of 0 to 4, with 0=no symptoms, 1=mild symptoms, 2=moderate symptoms, 3=more severe symptoms, and 4=severe symptoms (possible range 0- 20 or 24, for CANDLE and other interferonopathy patients, and SAVI respectively). At each visit, the diary score was calculated as follows:

a. Average score of each symptom was calculated using data entered since the previous visit and correcting for any day for which diary scores were not recorded.
b. The calculated average score for each symptom was summed up and divided by the number of assessed symptoms (i.e. 5 symptoms for CANDLE, 6 symptoms for SAVI) to calculate the average score for each patient.

Steroid reduction: An average diary score <0.5 (CANDLE diary) or <1.0 (SAVI diary) was indicative of a response to treatment and was one criterion used to initiate steroid weaning (if the patient was receiving steroids). Additionally, if the patient was responding to treatment, but did not meet the average diary score threshold to begin steroid weaning, but was experiencing new or worsening clinically significant adverse effects from steroids (including, but not limited to, cataracts, vertebral fractures due to osteoporosis, Cushingoid habitus, substantial weight gain, avascular necrosis, dyslipidemia, hypertension, opportunistic infections or stunted growth), the steroid weaning was permitted. Mean corticosteroid doses were calculated as prednisone equivalents in milligram (mg) per kilogram (kg) per day for each patient at each visit (http://www.medcalc.com/steroid.html)

Assessment of Remission*: The duration of remission for patients achieving remission criteria (DDS<0.15, off steroids, and CRP<5mg/L) was assessed by comparing the number of visits that patients fulfilled remission criteria for the visits before and after they first achieved remission criteria.

Disability and Quality of Life Assessments*: Questionnaires assessing disability and quality of life including Childhood Health Assessment Questionnaire (CHAQ), Pediatric Quality of Life Inventory (PedsQL), and physician and patient or parent global assessment (visual analog scale) were all completed at previous NIH visits or at the baseline visit, except for two patients (patient S2 and C10), who completed the questionnaires within 1 or 8 months of baricitinib initiation respectively. Questionnaires were obtained at most follow-up visits.

Height, weight, body mass index (BMI)*, bone age*, bone mineral density by Dual-energy x-ray absorptiometry (DEXA) measurements*, and Z-score calculations: Z-scores for height, weight, BMI and bone age were calculated as indicated in the respective table and figure legends. Patients with open growth plates at entry were considered to have growth potential. These patients were followed longitudinally. Z-scores for bone mineral density were calculated using the Bone Mineral Density in Childhood Study (BMDCS) calculator (https://bmdcs.nichd.nih.gov/zscore.htm) for patients between 2 and 19 years at the time of enrollment, Z-scores were adjusted for height. For patients older than 19 years, the NIH reported Z-scores were used. All patients had DEXA scans at the baseline visit or within 6 months of treatment initiation (except for two patients, patients C1 and C6 who obtained DEXAs 1 and 2.7 years post baricitinib respectively) that were used as "baseline". DEXA scans were repeated yearly as clinically indicated.

C. DISEASE-SPECIFIC OUTCOMES

CANDLE-specific outcomes:

The outcomes and measurements marked with an asterisk (*) were collected under the natural history protocol or are part of routine care of patient with chronic inflammation who are or are not receiving corticosteroids.

*Assessment of Lipid Profile**. Total cholesterol (TC), high-density lipoprotein cholesterol (HDL-C), low-density lipoprotein cholesterol (LDL-C) and triglycerides (TG) were obtained at baseline and at every visit (see Table S8). Hyperlipidemia is defined as high LDL and/or high TGs. NIH laboratory references were used for normality https://cc-internal.cc.nih.gov/LTGRA/UL/public_labtest_detail.

Assessment of Hepatic Steatosis by conventional In-Phase and Opposed-Phase MRI:* Liver fat was detected based on the relative signal loss on opposed-phase ("out-of-phase") images. Abdominal magnetic resonance imaging (MRI) were done every 6 to 12 months after treatment initiation.

Assessment of Myositis by MRI:* Six CANDLE patients had MRIs within 13 months of baricitinib initiation that confirmed clinical suspicion of myositis with characteristic muscle enhancement on MRI.

SAVI-specific outcomes:

*Chest Computed tomography (CT) scoring**: 20 Chest CT images from 4 SAVI patients collected at baseline and throughout the duration of treatment were scored by one radiologist (LF), who was blinded to the clinical data and the order of the scans. Inflammatory and lung damage findings were scored based on severity, with absent=0, mild or barely perceptible=1, moderate or obvious=2, severe or striking=3. *Inflammatory findings* that were scored included: 1. presence of ground glass opacities, 2. intralobular septal thickening, 3. pulmonary nodules, 4. consolidation, 5. atelectasis, 6. pleural effusion and 7. lymph nodes. *Lung damage findings* that were scored include: 1. presence of pneumothorax, 2. bronchiectasis, 3. parenchymal cyst(s), 4. subpleural cyst, and 5. vascular calcifications. Both lungs were scored together, the scores for the 7 inflammatory categories were added, the possible range of the inflammatory score is 0 (no abnormalities) to 21 (most severe score for all seven categories). The possible range of the summary damage score is 0 (no abnormalities) to 15 (most severe score for all 5 categories). For statistical analyses, the baseline CT scores were compared to the CT scores obtained at last visit included in the analysis up to February 2017.

D. IMMUNOLOGICAL ASSESSMENTS

1. *Inflammatory markers**. Acute phase reactants including high sensitivity (hs) C-reactive protein (CRP) and erythrocyte sedimentation rate (ESR), were collected at each visit under the NIH Natural History Protocol and compared to pre-treatment values.
2. *Hematologic values*. Hemoglobin (Hgb), white blood cell count (WBC), platelet count (Plts), and absolute lymphocyte count (ALC) were assessed at each visit. In patients with cytopenias at baseline, improvement of the cytopenias was an outcome measure. These parameters were also monitored for drug safety in all patients.
3. *Autoantibody titers, lymphocyte subset panel and immunoglobulin levels**. Anticardiolipin (ACA), anti-cyclic citrullinated peptide (anti-CCP), antinuclear (ANA), anti-double stranded DNA (anti-dsDNA); anti-myeloperoxidase (anti-MPO), anti-proteinase 3 (anti-PR3), antibody to extractable nuclear antigens (anti-ENA) and rheumatoid factor (RF) antibodies, as well as lymphocyte classes including T-cells, B-cells natural killer cells (NK) and monocyte counts and immunoglobulin levels (IgG, IgA and IgM) were monitored sporadically throughout the program.
4. *STAT-1 phosphorylation assay*.* For the STAT-1 phosphorylation assay, blood samples were collected in the mornings, before administration of the morning dose of baricitinib from 8 CANDLE and 4 SAVI patients. Healthy controls who did not receive baricitinib had blood draws for comparison. Whole blood was stimulated with cytokines IFN-α(PeproTech, Rocky Hill, NJ) for the assessment of STAT-1 phosphorylation. Median fluorescence intensity (MFI) of STAT-1 phosphorylation in CD4+ and CD8+ T cells, B cells and monocytes was measured by flow cytometry as previously described Liu et al 2011). Ratios of stimulated vs. unstimulated MFIs were calculated. For baseline samples, historical data (Liu et al Arthritis Rheumatol. 2011 and Liu et al. NEJM 2014) that we generated prior to treatment with baricitinib, were re-blotted to allow for comparison with samples obtained while patients were on baricitinib treatment.
5. *25-gene IFN score determination*.* RNA was prepared from peripheral blood collected in PAXgene tubes (Qiagen). A nanostring assay with 25-interferon response gene score (manuscript in press) was assessed at baseline and at most follow up visits until October 31, 2016. Based on control data, a normal IFN score was defined as below 44 (cut off is 95%ile in healthy controls).
6. *Cytokine Analysis by Luminex*.* A 27-plex assay that included the IP-10 analyte was performed on serum samples from all patients that were collected until December 2015 (Thermo Fisher, Raleigh, NC).

E. SAFETY ASSESSMENTS

1. _Assessment of lipid levels and lipid ratios._ Total/HDL and LDL/HDL cholesterol ratios are risk indicators with greater predictive value than isolated parameters used independently, particularly LDL. To assess cardiac risk, the TC/HDL-C ratio, the TG/ HDL-C ratio, and the LDL-C/H LDL-C ratios were calculated for each patient. (see Supplemental Table 8).

2. _BK titer assessment in blood and urine by polymerase chain reaction (PCR) assay_* were first assessed in the context of clinical care in the first identified patient (C7) in June 2015. When BK viral copies were elevated in the blood and urine, BK assessments were included in the routine safety evaluations under the Expanded Access program. BK titers in blood were retrospectively assessed in serum samples collected prior to baseline, at enrollment, and the different protocol visits, baseline urine samples were not collected and therefore baseline BK urine titers could not be obtained. Serial prospective Haufen testing as described below was also performed after identification of the first patient.

3. _Haufen Testing*_: Higher levels of BK viremia are associated with the development of BK nephropathy in renal transplant patients, but a definitive diagnosis requires a renal biopsy. To monitor whether the observed viruria and viremia lead to renal involvement and given the risks of an invasive kidney biopsy, we performed Haufen testing. The Haufen test detects aggregates of polyomavirus in urine and is proposed as a noninvasive biomarker for BK virus associated nephropathy (1). Briefly, 10-25 mL of urine were collected, fixed in a 1:1 ratio with 4% neutral-buffered paraformaldehyde, and stored at 4°C. To perform Polyoma Virus (PV)-Haufen testing, urine samples were sent to the University of North Carolina (2). Urine was concentrated and placed on an electron microscopy (EM) grid where the PV-Haufen adhere by electrostatic binding. One EM grid was prepared for each patient's urine sample and examined by transmission EM (LEO EM-910, LEO Electron Microscopy, Thornwood, NY; accelerating voltage, 90-100 kV at 80000-100000× magnification) for a maximum of 30 minutes, as previously described (1-3) by a pathologist who was blinded to each patient’s clinical information and who determined whether Haufen were present or absent. PV-Haufen are 3-dimensional, dense, cast-like, viral aggregates composed of 6 or more virions.

1. Singh HK, Andreoni KA, Madden V, et al. Presence of urinary Haufen accurately predicts polyomavirus nephropathy. J Am Soc Nephrol. 2009;20:416–427
2. Singh HK, Reisner H, Derebail VK, et al. Polyomavirus nephropathy: quantitative urinary polyomavirus-haufen testing accurately predicts the degree of intrarenal viral disease. Transplantation. 2015;99:609–615.
3. Singh HK, Donna Thompson B, Nickeleit V. Viral Haufen are urinary biomarkers of polyomavirus nephropathy: New diagnostic strategies utilizing negative staining electron microscopy. Ultrastruct Pathol. 2009;33(5):222-35

F. STATISTICAL ANALYSES

Per protocol, if more than 5 patients were enrolled into this program, the protocol defined that “…2-dimensional plots of various data may be utilized to explore the relationship between variables of interest including final dose level versus efficacy measures to explore recommended dosing guidelines, and plots of efficacy measures versus laboratory measures to explore risk/benefit relationships.”

Assessment of the duration of remission. 2x2 tables were calculated for the number of visits that each measure (steroid dose = 0 mg, DDS < 0.15 and hsCRP < 5mg/dL) fulfilled remission criteria and were compared before and after patients first reached remission criteria (two periods). The Cochran–Mantel–Haenszel test was used to analyze the association between these binary outcomes across patients.

I. Supplemental Tables

Supplemental Table 1: Baricitinib initial dosing, time to reach optimal tolerated doses

Subject number by disease	Weight at baseline (kg)	eGFR at baseline (mL/min/1.73m ^{2})	Weight Group	Date of first baricitinib dose	Start dose for baricitinib/ day (mg)	Last visit included in data analysis 10/31/2016	Time on program (days)	Date starting optimal dose	Time to reach optimal dose (days)	Time on optimal doses until data analysis (days)	Dosing regimen at time of optimal dose (mg/day)	Frequency	mg per dose
CANDLE 1	18.6	129.04	< 20 kg	10/26/11	0.10	9/19/16	1790	12/6/12	407	1383	6	BID	3mg/3mg
CANDLE 2	24.2	138.33	20-40 kg	2/28/12	0.20	10/11/16	1687	7/11/13	499	1188	6	BID	3mg/3mg
CANDLE 3	25.2	200.03	20-40 kg	7/11/12	1.00	9/19/16	1531	11/15/12	127	1404	6	BID	3mg/3mg
CANDLE 4	44.5	172.14	>40 kg	8/10/12	1.00	10/27/16	1539	2/4/13	178	1361	8	BID	6mg/2mg
CANDLE 5	65.5	101.76	>40 kg	9/21/12	1.00	10/17/16	1487	2/25/13	157	1330	9	BID	7mg/2mg
CANDLE 6	13.1	79.19	< 20 kg	3/8/13	1.00	6/6/16	1186	12/14/13	281	905	4	BID	2mg/2mg
CANDLE 7	11.6	82.8	< 20 kg	3/12/13	1.00	6/1/15	811	12/5/13	268	543	4	BID	2mg/2mg
CANDLE 8	13.4	219.9	< 20 kg	6/4/13	1.00	10/3/16	1217	10/22/13	140	1077	6	TID	2mg/2mg/2mg
CANDLE 9	43.2	138.31	>40 kg	9/27/13	3.00	9/6/16	1075	2/4/14	130	945	10	BID	5mg/5mg
CANDLE 10	41.3	166.4	>40 kg	3/24/14	7.00	9/12/16	903	8/20/14	149	754	9	BID	5mg/4mg
SAVI 1	25.8	172.73	20-40 kg	2/19/14	2.00	10/17/16	971	7/10/14	141	830	6	BID	3mg/3mg
SAVI 2	84.3	144.09	>40 kg	3/20/14	7.00	8/29/16	893	3/24/14	4	889	10	BID	7mg/3mg
SAVI 3	23.4	269.83	20-40 kg	5/13/14	3.00	10/24/16	895	5/19/14	6	889	6	BID	3mg/3mg
SAVI 4	23.6	189.34	20-40 kg	2/6/15	3.00	8/15/16	556	2/13/15	7	549	6	BID	3mg/3mg
OTHER 1	50.3	128.3	>40 kg	5/1/12	0.50	12/31/12	244	10/15/12	167	77	8	BID	6mg/2mg
OTHER 2	9.2	215.88	< 20 kg	7/15/13	1.00	8/15/16	1127	10/17/13	94	1033	6	TID	2mg/2mg/2mg
OTHER 3	50.0	76.42	>40 kg	8/21/13	3.00	10/21/13	61	8/26/13	5	56	5	QD	5mg
OTHER 4	49.8	186.76	>40 kg	2/18/14	3.00	5/23/16	825	10/1/14	225	600	9	BID	5mg/4mg

Individual listings of weight, eGFR, initial dosing, time on the program, date when optimal tolerated dosing was achieved and final dosing regimen.
eGFR - estimated glomerular filtration rate.
QD -once a day dosing, BID - twice a day dosing and TID - three times a day dosing.

Supplemental Table 2: Percent completion of diary data for all patients

Patient ID	Duration (in days) between Diary Dates	Complete Count	Complete %	Some Missing Count	Some Missing %	All Missing Count	All Missing %
C1	1805	1710	94.7%	15	0.8%	80	4.4%
C2	1700	1600	94.1%	15	0.9%	85	5.0%
O1	258	236	91.5%	0	0.0%	22	8.5%
C3	1546	1495	96.7%	8	0.5%	43	2.8%
C4	1557	1486	95.4%	21	1.3%	50	3.2%
C5	1506	1497	99.4%	5	0.3%	4	0.3%
C6	1331	1261	94.7%	6	0.5%	64	4.8%
C7	833	808	97.0%	3	0.4%	22	2.6%
C8	1236	1147	92.8%	12	1.0%	77	6.2%
O2	1226	1142	93.1%	16	1.3%	68	5.5%
O3	90	69	76.7%	0	0.0%	21	23.3%
C9	1088	1076	98.9%	0	0.0%	12	1.1%
O4	931	878	94.3%	14	1.5%	39	4.2%
S1	993	974	98.1%	2	0.2%	17	1.7%
C10	923	862	93.4%	55	6.0%	6	0.7%
S2	910	908	99.8%	0	0.0%	2	0.2%
S3	910	867	95.3%	5	0.5%	38	4.2%
S4	652	610	93.6%	36	5.5%	6	0.9%

Duration indicates the number of days a patient has been on the program at time of analysis.

Complete Count indicates the number of days when diary entries were made.

Complete% denotes the percentage of days a diary entry was made.

Some Missing Count indicates the number of days when diary entries were missing one or more components of the DDS.

Some Missing % indicates the percentage of incomplete DDS entries.

All Missing Counts indicates the number of days without any diary entry.

All Missing % indicates the percentage of days without any diary data.

Supplemental Table 3: Summary of primary outcomes (diary score and steroid dose), and on achieving clinical remission

Patient ID	Primary benefit[A] (DDS) response	Secondary benefit[B] (corticosteroids) response	Remission[C]
C1	No	**Yes**	
C2	Yes	Yes	**Yes**
O1[D]	No	No	
C3	Yes	Yes	
C4	Yes	Yes	**Yes**
C5	Yes	Yes	**Yes**
C6	Yes	Yes	
C7	No	No	
C8	Yes	Yes	
O2	Yes	Yes	
O3[D]	No	No	
C9	Yes	Yes	**Yes**
O4	No	**Yes**	
S1	No	No*	
C10	Yes	N/A	**Yes**
S2	Yes	N/A	
S3	Yes	N/A	
S4	Yes	N/A	
**CANDLE (n=10)**	8/10 (80%)	8/9 (89%)	5/10 (50%)
**SAVI (n=4)**	3/4 (75%)	0/1 (0%)	0/4 (0%)
**Other[D] (n=4)**	1/4 (25%)	2/4 (50%)	0/4 (0%)
**All (n=18)**	12/18 (67%)	10/14 (71%)	5/18 (28%)

[A] Diary score reduction criteria are a mean daily diary score of <0.5 for CANDLE and other IFNopathy, or <1 for SAVI.

[B] Prednisone reduction criteria is at least 50% decrease from baseline or < 0.15mg/kg/day. All CANDLE and other IFNopathy patients, except one (C10) were on oral corticosteroids at baseline, 1 SAVI pt. (S1) was on prednisone as well.

[C] Remission Criteria: 5 CANDLE patients (C2, C4, C5, C9, and C10) achieved remission criteria at their last visit with a mean diary score < 0.15, no prednisone, and a CRP < 5 mg/L. All CANDLE patients who achieved remission had mutations in PSMB8 the inducible proteasome components that are constitutively expressed in blood cells but are inducible in non-hematopoietic cells. The other 3 CANDLE patients (C3, C6 and C8) with digenic disease, fulfilled criteria for improvement in diary scores and had decreased steroid doses. Patient C1, who is compound heterozygous for *PSMB4* did not fulfilled criteria for improvement in diary score, he continues to be on prednisone doses of 0.27 mg/kg/day which was decreased from 0.84mg/kg/d at baseline. Pt. C7 developed BK viremia and azotemia and was discontinued from the program.

[D] 2 patients (pts.) (O1 and O3) discontinued after 244 and 98 days on the program (77 and 56 days on optimal tolerated dose). Pt. O1 discontinued due to lack of efficacy and Pt. O3 discontinued due to osteonecrosis; he also had an unsatisfactory response to treatment.

Supplemental Table 4: Stable and continued remission in 5 patients with CANDLE who achieved remission criteria on baricitinib treatment

		C2	C4	C5	C9	C10	p-value*	% visits on remission criterion
PRE-REMISSION	# NIH visits before remission	22	20	22	16	12		
	Off steroids	1/20	2/18	0/21	1/16	NA		5.3%
	DDS ≤0.15	0/22	18/20	13/22	9/16	6/12		50.0%
	CRP <5 mg/L	6/22	5/19	15/21	7/12	2/11		41.2%
	IFN score <42	5/14	3/14	1/4	3/4	0/4		30.0%
	Time before 1st remission (days)	1077	717	856	535	280	*Total: 3465*	
POST-REMISSION	Date of first remission	2/9/15	7/28/14	1/25/15	3/16/15	12/29/14		
	# of NIH visits since first remission	7	10	8	7	8		
	Remission	6/7	8/10	8/8	5/7	5/7		82.1%
	Off steroids	7/7	10/10	8/8	7/7	NA	<0.0001	100.0%
	DDS ≤0.15	6/7	10/10	8/8	5/7	7/8	<0.0001	90.0%
	CRP <5 mg/L	5/7	8/10	8/8	6/7	6/7	<0.0001	84.6%
	IFN score <42	5/7	7/9	3/4	3/4	0/3	0.0025	66.7%
	Time from 1st remission (days)	610	823	631	540	623	*Total: 3227*	

* P-value based on Cochran-Mantel-Haenszel statistical test comparing proportions before and after remission with the 5 patients combined.
All patients were in optimal tolerated doses of baricitinib at the time of remission.
The cut off for a normal 25-gene IFN score was 42 at the 95th % for healthy controls.
No patient restarted corticosteroids and fulfilled remission criteria at 82.1% of their follow up visits.

Supplemental Table 5: Changes in height Z-scores on baricitinib

Height Assessment	Pre-baricitinib Z-scores (Mean ± SD)	Post-baricitinib Z-scores (Mean ± SD)	Pre-baricitinib Mean height percentile (%) (Mean ± SD)	Post-baricitinib Mean height percentile (%) (Mean ± SD)	p-value[A]
**n=13**	-4.03 ± 2.64	-3.19 ± 2.33	1.46 ± 2.73	5.31 ± 11.97	0.4
**n=11[B]**	-4.10 ± 2.76	-3.02 ± 2.28	1.36 ± 2.84	6.0 ± 12.96	0.053
**n=9[C]**	-4.34 ± 3.0	-2.83 ± 2.42	1.4 ± 3.13	7.22 ± 14.17	***0.015***

Pre-baricitinib refers to baseline values that were collected prior to starting baricitinib. At the time of the last measurement, patients were on optimal tolerated doses of baricitinib for at least 1.5 years (excluding patients O1 and O3).
Clinical significant improvement in the height Z-scores and percentiles of patients with growth potential (n=13) was seen, when comparing pre-baricitinib to last visit on baricitinib data. Mean height Z-scores improved from -4.03 ± 2.64 to -3.19 ± 2.33; with "catch up growth" observed in 9 patients, their improvement translates into a mean height percentile increase from the 1.4th percentile to 7.2th percentile. When 4 patients, 2 who discontinued from the program (O1 and C7) and 2 who were not able to wean steroids to below 0.16 mg/kg/day (C1 and S1) were excluded, the change in Z-score became more significant.
[A] Indicates comparison between baseline to last clinic values, 2-sided *p-value* are denoted
[B] Analysis of data excluding two patients (O1 and C7) who discontinued from the program.
[C] Analysis of data excluding four patients (O1, C7 who discontinued and C1, and S1); all were unable to wean steroids to below 0.16 mg/kg/day

Supplemental Table 6: Changes in Dual-energy x-ray absorptiometry (DEXA) Z-scores on baricitinib treatment

DEXA scans n=15		Pre-baricitinib[A] Bone mineral density Z-score (Mean ± SD)	Post-baricitinib Bone mineral density Z-score (Mean ± SD)	p-value[B]
**Osteoporosis**	**n=8**	-4.64 ± 1.67	-3.04 ± 1.18	***<0.01***
**Osteopenia**	**n=2**	-2.15 ± 0.21	-1.45 ± 0.21	***<0.01***
**Normal Z-scores**	**n=5**	-1.45 ± 0.38	-1.15 ± 0.96	ns

[A] At least 2 DEXA scans were available for 15 patients, a second DEXA scan was missing on the 3 patients who discontinued treatment, C7, O1 and O3. For 11 patients DEXA scans were obtained prior to or within 4 weeks of baricitinib initiation. Patients C2, C3, S1 and S2 had DEXAs within 5 months prior to baricitinib initiation, and pt. O4, 9 months prior to baricitinib initiation. Two patients (C4 and C9) had their first DEXA scan within 6 months of treatment, and two other patients (C1 and C6), at 1 and 2.7 years after treatment initiation respectively. Height adjusted Z-scores were used for all except for pt. C8, a developmentally severely delayed pre-pubertal girl with a chronological age of 14.3 years and a mean height and bone age of 2 years, in her, we used the bone age for Z-score calculation. The site of the worst Z-score was used for comparisons (n=8 left femoral neck, n=5 AP spine (L1-L4) and n=2 total hip). At the time of their first DEXA scan, 8 out of 15 patients (53%) had osteoporosis (Z-score less than -2.5) with a mean Z-score of -4.64 ± 1.67; 2 out of 15 patients (13%) were osteopenic (Z-scores between -2.5 and -2.0) with a mean Z-score of -2.15 ± 0.21 and 5 patients (33%) had normal Z-scores. Three patients (C1, S1 and O4) with osteoporosis were on bisphosphonates, pt. O4 was started 30 days prior to baricitinib, patients S1 and C1, 11 and 24 months after starting baricitinib treatment.
[B] Comparison between first available DEXA vs. last visit, patients with osteoporosis and osteopenia were grouped for this analysis. *p-values* were not adjusted.

Supplemental Table 7: Changes in Body Mass Index (BMI) on baricitinib

Body Mass Index[A] (BMI) n =18	Pre-baricitinib n (%)	Post-baricitinib n (%)
**Obesity**	7/18 (39%)	3/18 (17%)
**Overweight**	1/18 (6%)	6/18 (33%)
**Normal BMI**	5/18 (28%)	6/18 (33%)
**Underweight**	5/18 (28%)	3/18 (17%)

7 pediatric patients who were all on steroids (C3, C5, C6, C7, S1, O1 and O4) had BMI > 95% at baseline, meeting the Centers for Disease Control and Prevention (CDC) criteria for obesity, 1 adult patient (S2) had a BMI of 28.5 meeting the CDC criteria for overweight. 5 patients (C9, C10, S3, S4 and O3) were considered underweight with BMI < 18.5 or < 5% for pediatric patients. The remainder 5 patients had normal BMI (C1, C2, C4, C8 and O2). At the last visit, 5 out of 7 obese patients had a significant clinical reduction in their BMI (C3, C5, C6, S1, and O4 are now overweight), 2 patients did not have changes on their BMI (C7 and O1 - both discontinued participation and both were unable to wean steroids). The BMI in patient S2 increased now meeting obesity criteria. 4 out of 5 underweight patients (C9, C10, S4 and O3) had significant clinical improvement in their BMI, 2 patients (C9 and C10) normalized their BMI and 2 patients (S4 and O3) with improvement remain in the underweight range. One of them, Pt. S4 with the lowest BMI had a baseline Z-score of -9 (not shown). All patients with normal BMIs continue to have normal BMIs, except for the youngest patient who has a current BMI at the 87th percentile and is overweight.
[A] BMI percentiles were calculated for patients 2 to 20 years.
Obesity: BMI ≥ 95th percentile (pediatrics), or ≥ 30 (adults)
Overweight: BMI ≥ 85th to < 95th percentile (pediatrics), or ≥ 25 to < 30 (adults)
Normal: BMI ≥ 5th to < 85thpercentile (pediatrics), or ≥ 18.5 to < 25 (adults)
Underweight: BMI < 5th Percentile (pediatrics), or < 18.5 (adults)

Supplementary Table 8: Changes in lipid profile and lipid ratios on baricitinib treatment

Lipid Profile n=18	Pre-baricitinib (Mean ± SD)	Post-baricitinib (Mean ± SD)	Pre-baricitinib (Median) (Interquartile range)	Post-baricitinib (Median) (Interquartile range)	p-value[A]
**Total Cholesterol (TC) (mg/dL)**	162.72 ± 46.07	194.28 ± 57.50	148.00<br>130.75 - 187.00	185.50<br>151.50 - 224.00	***<0.01***
**HDL-C (mg/dL)**	38.50 ± 17.62	52.28 ± 18.23	32.50<br>26.25- 49.00	51.50<br>37.75 - 68.00	***0.001***
**LDL-C (mg/dL)**	95.56 ± 37.50	111.44 ± 43.38	82.00<br>69.25 - 113.00	103.00<br>77.50 - 137.50	0.056
**Triglycerides (TG) (mg/dL)**	184.89 ± 199.67	152.83 ± 91.43	109.00<br>94.00 - 187.75	126.50<br>76.75 - 181.00	ns

Lipid ratios n=18	TC/HDL-C Risk ratio > 4.0 Pre - Post n (%)	LDL-C/HDL-C Risk ratio > 2.5 Pre – Post n (%)	TG/HDL-C Risk ratio >3 Pre – Post n (%)
**All patient n=18**	11/18 (61%) - 8/18 (44%)	11/18 (61%) - 8/18 (44%)	10/18 (56%) - 8/18 (44%)
**CANDLE patients[B] n=10**	8/10 (80%) - 6/10 (60%)	8/10 (80%) - 6/10 (60%)	7/10 (70%) - 6/10 (60%)
**SAVI patients n=4**	1/4 (25%) - 1/4 (25%)	1/4 (25%) - 1/4 (25%)	1/4 (25%) - 1/4 (25%)
**Other IFNopathy patients n=4**	2/4 (50%) - 1/4 (25%)	2/4 (50%) - 1/4 (25%)	2/4 (50%) - 1/4 (25%)

Total cholesterol (TC), high-density lipoprotein cholesterol (HDL-C), and low-density lipoprotein cholesterol (LDL-C) increased on baricitinib treatment. Of interest, TC/HDL-C ratio improved on baricitinib. As there are no pediatric references for lipid ratios, we used target risk ratios for primary prevention in women TC/HDL-C ratio <4.0 and LDL-C/HDL-C ratio <2.5 and triglycerides TG/HDL-C ratio <3.0 as cut off values for the assessment (see references below).

[A] Pre-baricitinib values were compared to post baricitinib values (last visit), *p-value* were not adjusted.

[B] Only patients C1 and C3 normalized their lipid ratios, both received fibrates early on in the course of the program.

References:

1. Millan J, Pinto X, Muñoz A, et al. Vasc Health Risk Manag. 2009; 5: 757–765
2. Gotto AM, Assmann G, Carmena R, et al. The ILIB lipid handbook for clinical practice: blood lipids and coronary heart disease. 2nd ed. New York, NY: International Lipid Information Bureau; 2000. p. 52.p. 53.p. 201.
3. Genest J, Frohlich J, Fodor G, et al. CMAJ. 2003;169:921–924.
4. Holme I, Aastveit AH, Junger I, et al. J Intern Med. 2008;264:30–38.
5. McLaughlin T, Abbasi F, Cheal K, et al. Ann Intern Med. 2003;139:802-809

Supplemental Table 9: Changes in muscle enzymes in CANDLE patients

Muscle Enzymes in CANDLE patients n=10	Pre-baricitinib (Mean ± SD)	Post-baricitinib (Mean ± SD)	Pre-baricitinib (Median) (IQR)	Post-baricitinib (Median) (IQR)	p-value[A]
**Aldolase (U/L)**	19.64 ± 14.30	9.36 ± 3.51	15.50<br>11.30 - 21.18	9.65<br>6.08 - 12.63	*0.06*
**Creatinine Kinase (U/L)**	536 ± 842.11	241 ± 145.56	134<br>55 - 303	199<br>177.25 - 275.50	ns
**Lactate dehydrogenase (U/L)**	413.33 ± 245.36	357.20 ± 391.68	384<br>278 - 531	224<br>171.75 - 326	ns
**Alanine Transferase (U/L)**	45.10 ± 21.35	47.40 ± 36.59	41.50<br>30.50 - 62	35<br>22 - 50.50	ns
**Aspartate Aminotransferase (U/L)**	57.30 ± 44.66	41.30 ± 18.02	41.50<br>30.50 - 60.25	39<br>24 - 57.50	ns

Myositis is a common feature in CANDLE patients; 8 of the 10 CANDLE patients had a history of myositis prior to enrollment. In 6 patients, myositis was demonstrated on MRI (4 patients had an MRI at baseline, and 2 patients within 13 months after baricitinib initiation). Muscle enzymes including creatinine kinase (CK), lactate dehydrogenase (LDH), aldolase, alanine transferase (ALT) and aspartate aminotransferase (AST), were assessed at each visit. ALT and AST improved in all CANDLE patients, except two, patient C4 who developed hepatic steatosis and patient C9 who had transient elevation of liver enzymes at the last visit, probable related to the use of protein supplements at that visit. After discontinuation of supplements LFTs normalized. Aldolase and LDH levels also decreased. CK levels however increased. At the last visit, 6 out of 10 patients had an elevation of the creatinine kinase without clinical evidence of myositis. The cause for CK elevations is likely multifactorial, CK elevations are also seen in SAVI patients who do not have myositis, and may represent an increase in muscle mass due to improved growth and increase in physical activity, or as an effect of baricitinib treatment (see in safety section).

[A] Pre-baricitinib values were compared to post baricitinib values (last visit), *p-value* were not adjusted.

Supplemental Table 10: Changes in pulmonary function test and chest CT in SAVI patients

Lung physiologic features in SAVI patients (n=4)	Pre-baricitinib[A] (Mean ± SD)	Post-baricitinib (Mean ± SD)	P-value[B]
Pulmonary Function Test (PFTs)			
**FVC pre-lt**	1.90 ± 1.12	2.30 ± 1	<0.05
**FVC-% of predicted value**	64.75 ± 11.95	69.25 ± 5.74	ns
**FEV1 pre-lt**	1.57 ± 0.90	1.91 ± 0.79	<0.05
**FEV1 pre-% of predicted value**	59.75 ± 12.42	65 ± 5.35	ns
**TLC-lt**	3.09 ± 1.31	3.02 ± 1.25	ns
**TLC-% of predicted value**	77.33 ± 24.79	71.50 ± 10.79	ns
Diffusion Lung Capacity (DLCO)			
**Carbon monoxide diffusing capacity Adj mL/mmHg/min**	9.73 ± 7.02	12.73 ± 6.23	<0.001
**Carbon monoxide diffusing capacity Adj – % of predicted value**	46.75 ± 28.24	48 ± 16.39	ns
6 Minute Walk Test (6MWT)[C] n=3			
**Distance on 6-min walk test -m**	397.33 ± 77.26	425.33 ± 49.81	ns
**Dyspnea score -post**	2.33 ± 0.58	3.67 ± 1.53	ns
**Peripheral capillary oxygenation -post**	97.33 ± 0.58	96.33 ± 2.08	ns

High-resolution computed tomography score	Pre-baricitinib (Mean) (min-max)	Post-baricitinib (Mean) (min-max)	
**Damage score[D]**	2.25<br>(0 - 6)	2.25<br>(0 - 6)	ns
**Inflammatory score[E]**	3.25<br>(1 - 5)	3.25<br>(2 - 5)	ns

[A] All pulmonary function test (PFTs), diffuse lung capacity (DLCO) and 6-minute walk test (6MWT) were obtained prior or within 10 days of the first dose of baricitinib.
[B] Pre-baricitinib values were compared to post baricitinib values (last visit), *p-value* were not adjusted.
[C] Patients S3 and S4 were non-ambulatory and unable to complete the 6MWT at baseline. After 3 months on baricitinib, patient S4 was able to do his first 6MWT, this was considered baseline for this analysis.
[D] Damage score was based on the presence of pneumothorax, bronchiectasis, parenchymal cyst, subpleural cyst, and vascular calcifications, each category was scored as absent=0, mild or barely perceptible=1, moderate or obvious=2, severe or striking=3. Damage score range from 0-15.
[E] Inflammatory score was based on the presence of ground glass opacities, intralobular septal thickening, pulmonary nodules, consolidation, atelectasis, pleural effusion and lymph nodes. The inflammatory score ranges from 0-21.

The “worsening in post-dyspnea scores” likely reflects the fact that patients are now able to exert themselves and walk further, which they have not been able to do before treatment.

Supplemental Table 11: Change of antibody profile in CANDLE, SAVI and other IFNopathies on baricitinib

Patient ID	Antibody Positivity Yes/No	Antibody positive	Change on treatment
C1	Yes	LAC	Turned negative before study entry
C2	No		
C3	Yes	ANA	Turned negative
C4	Yes	LAC	Turned negative
C5	No		
C6	No		
C7	No		
C8	Yes	LAC, ACA IgM	Both stayed positive
C9	Yes	LAC, anti-MPO	Not retested. Stayed positive
C10	Yes	LAC	Turned negative
S1	Yes	ANA, ACA IgM, anti-SSA	ACA IgM and anti-SSA turned negative, ANA stayed positive
S2	Yes	ANA, LAC, anti-PR3, anti-dsDNA	All turned negative
S3	Yes	ANA, LAC, anti-PR3	All turned negative
S4	Yes	ANA, LAC, ACA IgG	ACA IgG turned negative. Other two antibodies remain positive
O2	Yes	LAC	Stayed positive
O4	Yes	LAC, ACA IgM	ACA IgM turned negative, LAC stayed positive
O1	ND		
O3	ND		

SAVI patients had more autoantibody positivity than patients with other IFNopathies and with CANDLE. Mean number of autoantibodies per patient decreases with baricitinib treatment across all disease groups (*p=0.013*). Anticardiolipin antibody (ACA) IgM, ACA IgM; anticardiolipin antibody (ACA) IgG, ACA IgG; antinuclear antibody, ANA; anti-double stranded DNA antibody, anti-dsDNA; anti-myeloperoxidase antibody, anti-MPO; anti-proteinase 3 antibody, anti-PR3; anti-Sjogren’s-syndrome related antigen A antibody, anti-SSA, lupus anticoagulant (LAC). Not done, ND.

Supplemental Table 12: Association of IFN biomarkers (IP-10 and 25-gene IFN score) with Clinical Outcomes and with historical biomarkers (ESR and CRP)

	DDS	Corticosteroids	25-IFN Score	IP-10	ESR
**Conventional biomarkers**					
**CRP**[A]	0.114	0.061	0.108	0.462	0.379
p-value[B]	*0.0078*	0.097	0.058	*0.025*	*<0.0001*
**ESR**	0.178	-0.127	0.251	0.312	
p-value	*0.0048*	0.210	*0.006*	*0.047*	
**IFN biomarkers**					
**IP-10**	0.097	0.13	0.367		
p-value	*0.0021*	*0.0024*	*0.0006*		
**25-IFN Score**	0.184	0.199			
p-value	*0.014*	*0.005*			

[A] correlation values are shown
[B] p-values of the slope from the linear mixed model analysis (see Statistical analysis of the Supplementary Methods) are denoted.
Correlations between IP-10, 25-IFN Score, the inflammatory disease markers (erythrocyte sedimentation rate (ESR) and C-reactive protein (CRP)) and the clinical outcomes (average daily diary score (DDS), and daily dose of steroids) were assessed.
While IFN biomarkers and conventional biomarkers correlated similarly with patients' symptoms, the IFN biomarkers correlated better with the ability to wean corticosteroids.

Supplemental Table 13: Serious Adverse Events (SAE) per patient[A]

Patient	SAE requiring hospitalization	SAE for other medically significant reason
C1	Failure to thrive, Osteoporosis, Nephrolithiasis (6 occurrences), Fluid replacement (IV hydration), Herpes Zoster[I]	BK virus infection (BK viremia)[B,K]
C3	none	BK virus infection (BK viremia)[B,K]
C5	none	BK virus infection (BK viremia)[B,K]
C6	CANDLE flare, Pyrexia (3 occurrences)[I], Influenza, Dehydration (2 occurrences), Device related infection (portacath infection), Urinary tract infection, Knee deformity[C], Pneumonia	none

C7 (patient discontinued due to acute kidney injury)	Polyomavirus test positive suggestive of BK virus infection (BK viremia and viruria), Clostridium difficile infection, Anemia[D], CANDLE flare (3 occurrences)[E], Dehydration, Haemophilus bacteremia, Influenza, Device-related infection (portacath infection), Pneumocystis jirovecii pneumonia (unconfirmed PCP), Viral infection, Rotavirus infection, Renal tubular disorder (3 occurrences), Acute kidney insufficiency (2 occurrences), Thrombocytopenia (2 occurrences)[G], Epistaxis[F], Pyrexia[F], Medical observation (during first dose of ruxolitinib)[F]	Neutropenia[E]
C8	none	BK virus infection (BK viremia)[B,K]
C9	Jaw operation (elective jaw surgery)	
C10	Herpes zoster	BK virus infection (BK viremia)[B,K]
O2	none	BK virus infection (BK viremia)[B,K]
O4 (patient with pre-existing Moya-Moya with a history of stroke S/P CNS vascular shunt surgery)	Cerebrovascular disorder (2 occurrences), Intracranial pressure increased (3 intermittent occurrences), Urinary tract infection[H], Ureterolithiasis, Pyelonephritis, Urosepsis (life-threatening), Clostridium difficile infection, Nephrolithiasis, Headache	Hepatic adenoma
O3[J] (patient with pre-existing nodular regenerative hyperplasia, portal hypertension and esophageal varices that required prior ligation. Patient discontinued due to osteonecrosis)	Upper gastrointestinal hemorrhage (2 occurrences of bleeding from previously ligated esophageal varices due to nodular regenerative hyperplasia and portal hypertension), Thrombocytopenia[D], Osteonecrosis	none
S1	Rash, Viral gastroenteritis	none
S2	Wound infection, Skin ulcer (necrotic ulcer toe), Extremity necrosis (finger), Wound (bone exposure finger)	none
S3	Pneumonia, Osteomyelitis (2 occurrences), Localized infection (feet – 2 occurrences), Wound infection	BK virus infection (BK viremia)[B,K]
S4	Hypoxia, Pneumonia (2 occurrences), Renal biopsy[L], Vasculitis	BK virus infection (BK viremia)[B], Positive Haufen test[I]

[A] Unless otherwise noted, the SAEs have resolved without interruption of baricitinib treatment.
[B] BK viremia has been intermittent and was reported as ongoing at the time of the analysis.
[C] Elective surgery for pre-existing bilateral genu valgum.
[D] Ongoing at the time of discontinuation from the program.
[E] Baricitinib had been temporarily suspended for 3 days due to an adverse event of pneumonia at the time of the SAEs of first CANDLE flare and neutropenia.
[F] Baricitinib had been permanently discontinued at the time of the SAE.
[G] Baricitinib had been permanently discontinued at the time of the SAE (thrombocytopenia 2nd occurrence).
[H] Baricitinib had been temporarily suspended due to an adverse event of neutropenia at the time of this SAE.
[I] Ongoing at the time of the analysis
[J] Pt. had pre-existing IgA nephropathy with glomerulosclerosis and renal insufficiency
[K] Negative Haufen testing

[L] Renal biopsy was negative for BK nephropathy in context of positive Haufen test

Supplemental Table 14: Treatment-emergent Categorical Laboratory Results of Interest

Categorical Laboratory Result	Number of Patients	Comment
eGFR <60 mL/min/1.73 m^2	2	**O3[A]:** had pre-existing IgA nephropathy and discontinued due to osteonecrosis<br>**C7[A]:** permanently discontinued the program due to acute kidney injury
ALT or AST >3X ULN with T.Bili >2X ULN	0	
Anemia (Hgb <6.5 g/dL)	0	
Leukopenia (WBC <2000 cells/µL)	0	
Neutropenia (ANC <1000 cells/µL)	3	**C2, O3[A]:** each patient met criteria on 1 occasion post-baseline which resolved to above criteria without interruption of baricitinib<br>**S1:** patient met criteria on 1 occasion post-baseline which resolved to above criteria with dose reduction
Lymphopenia (lymphocyte count <500 cells/µL)	2	**C7[A]:** met criteria on 4 occasions post-baseline which resolved to above criteria without interruption of baricitinib<br>**S3:** met criteria on 2 occasions post-baseline which resolved to above criteria without interruption of baricitinib
Thrombocytosis (platelet count >600,000/µL)	4	**O2:** elevated platelets at baseline that increased over time<br>**S1:** met criteria on 2 occasions post-baseline which resolved to below criteria with dose reduction<br>**S4:** met criteria on 1 occasion post-baseline which resolved to below criteria without interruption of baricitinib<br>**O4:** met criteria at last 2 post-baseline observations at time of analysis
Thrombocytopenia (platelet count <75,000/µL)	2	**O3[A]:** had pre-existing thrombocytopenia with low platelet count (36,000/µL) at time of discontinuation due to osteonecrosis<br>**C7[A]:** met criteria on 1 post-baseline occasion which resolved to above criteria with dose reduction prior to discontinuation due to acute kidney injury

[A] Patients who permanently discontinued due to adverse events

Supplemental Table 15. Treatment-emergent Adverse Events (non-infection) by System/Organ Group with Individual Event Terms Shown if in ≥4 Patients

Adverse Event	CANDLE Syndrome, n=10	CANDLE-related Conditions, n=4	SAVI, n=4	Total, N=18
	n (%)	n (%)	n (%)	n (%)
**Blood and lymphatic system disorders**	**6 (60.0)**	**1 (25.0)**	**2 (50.0)**	**9 (50.0)**
Anemia	3 (30.0)	0	1 (25.0)	4 (22.2)
Neutropenia	1 (10.0)	1 (25.0)	2 (50.0)	4 (22.2)
Thrombocytosis	2 (20.0)	1 (25.0)	1 (25.0)	4 (22.2)
**Cardiac disorders**	**3 (30.0)**	**0**	**1 (25.0)**	**4 (22.2)**
**Congenital, familial and genetic disorders**	**1 (10.0)**	**0**	**0**	**1 (5.6)**
**Ear and labyrinth disorders**	**2 (20.0)**	**1 (25.0)**	**1 (25.0)**	**4 (22.2)**
**Endocrine disorders**	**1 (10.0)**	**2 (50.0)**	**0**	**3 (16.7)**
**Eye disorders**	**3 (30.0)**	**0**	**0**	**3 (16.7)**
**General disorders and administration site conditions**	**4 (40.0)**	**1 (25.0)**	**1 (25.0)**	**6 (33.3)**
**Gastrointestinal disorders**	**7 (70.0)**	**3 (75.0)**	**2 (50.0)**	**12 (66.7)**
Diarrhea	4 (40.0)	2 (50.0)	0	6 (33.3)
Abdominal pain	3 (30.0)	0	2 (50.0)	5 (27.8)
**Hepatobiliary disorders**	**4 (40.0)**	**1 (25.0)**	**0**	**5 (27.8)**
Hepatic steatosis	3 (30.0)	1 (25.0)	0	4 (22.2)
**Injury, poisoning and procedural complications**	**8 (80.0)**	**1 (25.0)**	**3 (75.0)**	**12 (66.7)**
Fracture[B]	2 (20.0)	1 (25.0)	2 (50.0)	5 (27.8)
**Investigations**	**10 (100.0)**	**3 (75.0)**	**4 (100.0)**	**17 (94.4)**
Polyomavirus test positive (BK viruria)	10 (100.0)	2 (50.0)	3 (75.0)	15 (83.3)
Transaminases increased	6 (60.0)	2 (50.0)	1 (25.0)	9 (50.0)
Weight increased	4 (40.0)	1 (25.0)	3 (75.0)	8 (44.4)
Blood creatine phosphokinase increased	3 (30.0)	1 (25.0)	1 (25.0)	5 (27.8)
**Metabolism and nutrition disorders**	**9 (90.0)**	**3 (75.0)**	**4 (100.0)**	**16 (88.9)**
Vitamin D deficiency	4 (40.0)	0	2 (50.0)	6 (33.3)
Decreased appetite	2 (20.0)	0	2 (50.0)	4 (22.2)
Hypercholesterolemia	4 (40.0)	0	0	4 (22.2)
**Musculoskeletal and connective tissue disorders**	**7 (70.0)**	**2 (50.0)**	**4 (100.0)**	**13 (72.2)**
Back pain	4 (40.0)	1 (25.0)	0	5 (27.8)
**Neoplasms benign, malignant and unspecified (including cysts and polyps)[C]**	**1 (10.0)**	**1 (25.0)**	**0**	**2 (11.1)**
**Nervous system disorders**	**4 (40.0)**	**1 (25.0)**	**1 (25.0)**	**6 (33.3)**
Headache	4 (40.0)	1 (25.0)	1 (25.0)	6 (33.3)
**Psychiatric disorders[D]**	**3 (30.0)**	**0**	**0**	**3 (16.7)**

**Renal and urinary disorders**	**6 (60.0)**	**1 (25.0)**	**3 (75.0)**	**10 (55.6)**
Proteinuria	2 (20.0)	0	3 (75.0)	5 (27.8)
**Reproductive system and breast disorders**	**1 (10.0)**	**0**	**1 (25.0)**	**2 (11.1)**
**Respiratory, thoracic and mediastinal disorders**	**8 (80.0)**	**2 (50.0)**	**3 (75.0)**	**13 (72.2)**
Cough	5 (50.0)	1 (25.0)	3 (75.0)	9 (50.0)
**Skin and subcutaneous tissue disorders**	**7 (70.0)**	**1 (25.0)**	**4 (100.0)**	**12 (66.7)**
Acne	2 (20.0)	1 (25.0)	3 (75.0)	6 (33.3)
**Surgical and medical procedures**	**4 (40.0)**	**0**	**0**	**4 (22.2)**
**Vascular disorders**	**1 (10.0)**	**1 (25.0)**	**2 (50.0)**	**4 (22.2)**

Treatment-emergent adverse events are defined as those events that were new or that worsened after starting baricitinib treatment. Patients with multiple occurrences of a specific event are counted once for the event. Patients with multiple events within a grouped term are counted once for the group term.
[A] Cardiac disorders includes: palpitations, sinus bradycardia, tachycardia
[B] All patients with fractures had at least 1 risk factor including chronic use of systemic corticosteroids (4 of 5 patients), pre-existing osteoporosis (2 patients), or pre-existing osteopenia (1 patient).
[C] Neoplasms benign, malignant, and unspecified (including cysts and polyps) includes: acrochordon, angiomyolipoma, hepatic adenoma
[D] Psychiatric disorders includes: mood altered and attention deficit/hyperactivity disorder

Supplemental Table 16: BK virus titers initial vs. last included assessment

BK titers [Log10] n=15	Pre-baricitinib[A]	Post-baricitinib[B] June 2015 n (%) (Mean ± SD)	Post-baricitinib[C] February 2017 n (%) (Mean ± SD)	p-value[D]
**BK titers urine [log10]**	Not done	13/15 (87%) 5.85 ± 2.29	14/15 (93%) 6.10 ± 2.18	ns
**BK titers blood [log10]**	2/15 (13%)	3/15 (20%) 3.54 ± 1.84	7/15 (47%) 2.58 ± 0.72	ns

Results reported in [log10] copies/mL; n (%) represents the number and (percentage) of patients that tested positive for BK virus in urine or blood.
[A] After the identification of the initial patient (pt. C7), stored serum samples from 15 patients were screened for BK virus in blood and urine by PCR analysis. 2 patients (C3 and O4) were intermittently positive prior to the first dose of baricitinib, patient C3 was positive on two different occasions, 1.5 years and 4 months prior to enrollment, patient O4 was positive 10 months prior to first dose of baricitinib. The first case (C7) was identified on June 3, 2015 based on rising creatinine, tubular proteinuria and presence of decoy cells in the urine. BK titers at the time of assessment were 10.16 [log10] in urine, and 6.37 [log10] in blood.
[B] Post-baricitinib (June 2015) - Denotes time of identification of the first case (pt. C7)
[C] Post – baricitinib (February 2017) – BK data available at last NIH visit as of February 2017
[D] The first BK titer assessment at the time of the identification of the first case (post-baricitinib - June 2015) were compared to the last visit.
BK virus is ubiquitous and typically acquired in childhood.[1] The infection is typically asymptomatic and viral reactivation manifesting as asymptomatic viral shedding in the urine is not uncommon in immunocompetent individuals but increased in immunocompromised patients.[2-4] Symptomatic reactivation and the development of BK-associated nephropathy are seen in renal and hematopoietic stem cell transplant populations.[5] The presence of viral reactivation in our patients with immune dysregulation and exposure to long-term immunosuppression since childhood might therefore be expected but should be monitored until more data are available.

We collected 79 urine samples from 14 patients with BK viremia and/or BK viruria, samples were assessed between October 2015 and May 2017. Of the 79 urine samples evaluated, only one patient (S4) had a positive Haufen result, this was confirmed on 3 random urine samples collected over 8 days between from 8/12/16 to 8/20/16. The BK blood titers at the time of urine sample collection were 2.87 Log10 IU/mL (08/15/2016) and 3.03 Log10 IU/mL (08/19/2016) respectively, BK titers in urine were consistently elevated at >9.81 Log10 IU/mL. A renal biopsy was performed 66 days after a positive Haufen testing and after reducing baricitinib dose by 28%, histologic findings were not consistent with BK nephropathy. We subsequently assessed 3 urine samples from patient S4 within1 to 4 months after the kidney biopsy and all were negative for Haufen.

References:

1. Siguier M, Sellier P, Bergmann JF. BK-virus infections: a literature review. Med Ma Infect 2012;42:181-7.
2. Knowles WA. Discovery and epidemiology of the human polyomaviruses BK virus (BKV) and JC virus (JCV). Adv Exp Med Biol 2006;577:19-45.
3. Rinaldo CH, Tylden GD, Sharma BN. The human polyomavirus BK (BKPyV): virological background and clinical implications. APMIS 2013;121:728-45.
4. Polo C, Perez JL, Mielnichuck A, Fedele CG, Niubo J, Tenorio A. Prevalence and patterns of polyomavirus urinary excretion in immunocompetent adults and children. Clin Microbiol Infect 2004;10:640-4.
5. Zhou W, Sharma M, Martinez J, et al. Functional characterization of BK virus-specific CD4+ T cells with cytotoxic potential in seropositive adults. Viral Immunol 2007;20:379-88.

II. Supplementary Figures

Supplemental Figure 1. Patient enrollment and disposition

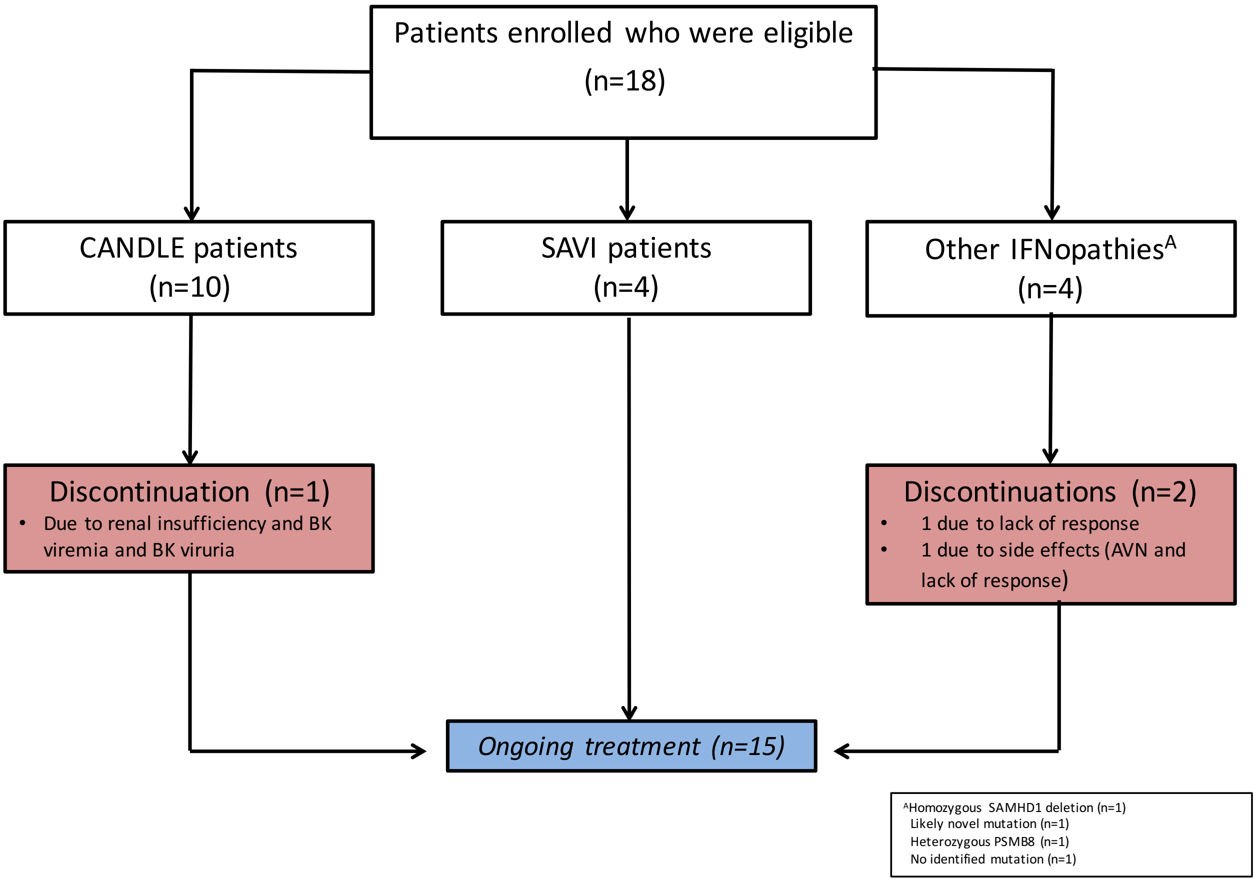

Three patients discontinued the program, one due to non-response to treatment, one due to osteonecrosis; he also had an unsatisfactory response, and one patient due to the development of renal insufficiency and BK viremia and BK viruria.

Supplemental Figure 2. Improvement in clinical disease manifestations in patients with CANDLE and other IFNopathies treated with baricitinib

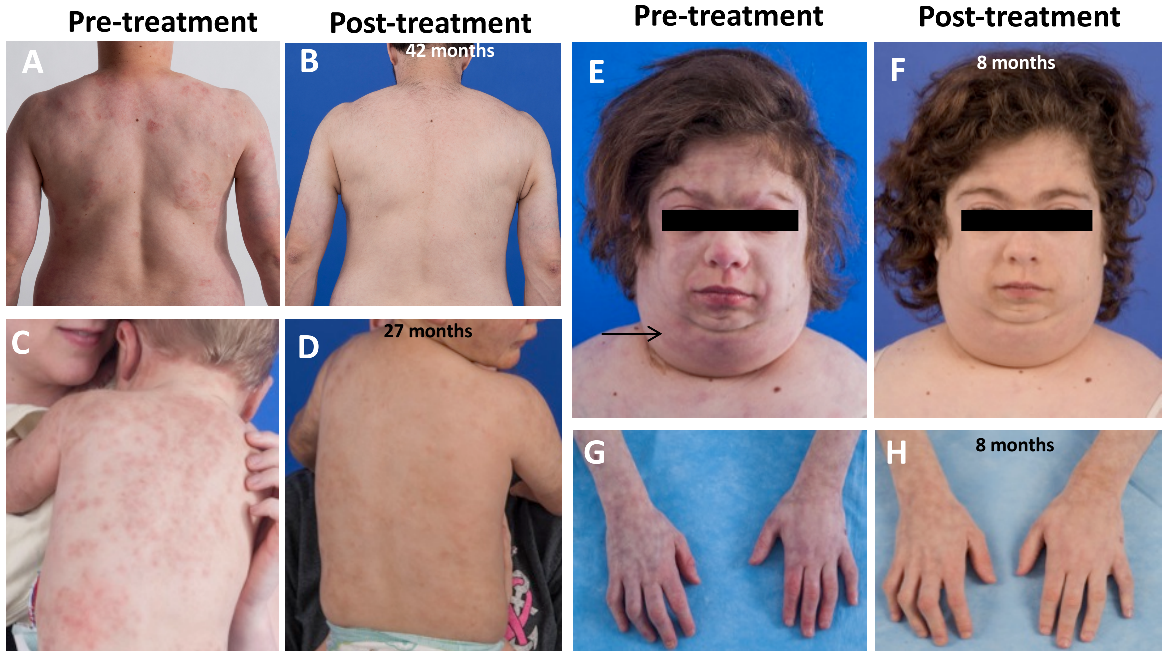

(A) (pre-treatment) CANDLE patient (C5) who achieved remission criteria. Images of back show classical distribution of rash. **(B) (post-treatment) shows** complete resolution of areas of panniculitis on lower back. There is no change in lipodystrophy. **(C, D)** Patient with a yet uncharacterized novel interferonopathy, image shows improvement of urticarial papules on upper and lower back, patient continues with significant skin rashes and elevated acute phase reactants. **(E-H)** Patient homozygous for *SAMHD1* deletion (O4). Images of face, upper chest and bilateral hands shows improvement of livedo reticularis as well as decreased areas of erythema over subcutaneous calcifications (Panel E, F area of black arrow).

Supplemental Figure 3. Delay in Bone Age at baseline

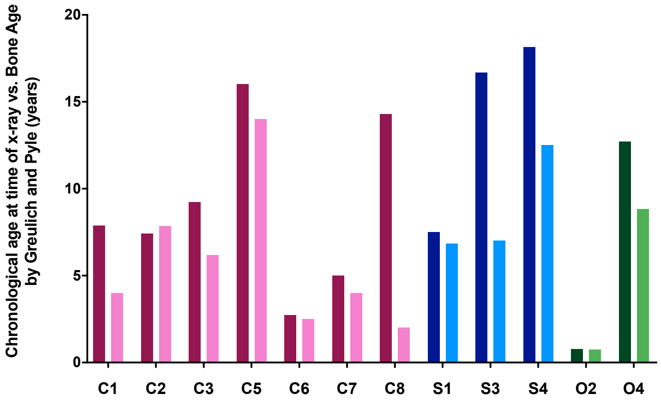

Bone Age (n=12)	Chronological Age (Years)	Bone Age by Greulich and Pyle (Years)	Bone Age Delay (Years)	p-value
**Mean (SD)**	9.86 ± 5.67	6.37 ± 4.06	3.49 ± 3.99	***<0.05***

Bone Age by Greulich and Pyle was compared to chronological age at time of x-ray. Mean delayed bone age was 3.49 ± 3.99 years ($p < 0.05$). All patients with growth potential were included except for patient O1, no bone age was available for this patient (n=12). X-rays were obtained within 6 months of baricitinib initiation, except for two patients (C3 and C7), their bone age was assessed 3 years and 17 months after program initiation respectively.

Supplemental Figures 4A-E. Anthropometric measurements in patients with CANDLE without growth potential (C4, C9 and C10), and with growth potential (C2, C3, C5, and C1, C6, C7 and C8), in patients with SAVI and with other IFNopathies

Baricitinib First Dose | Baricitinib Optimal Dose | Growth Hormone Initiated | Baricitinib Discontinued

A.

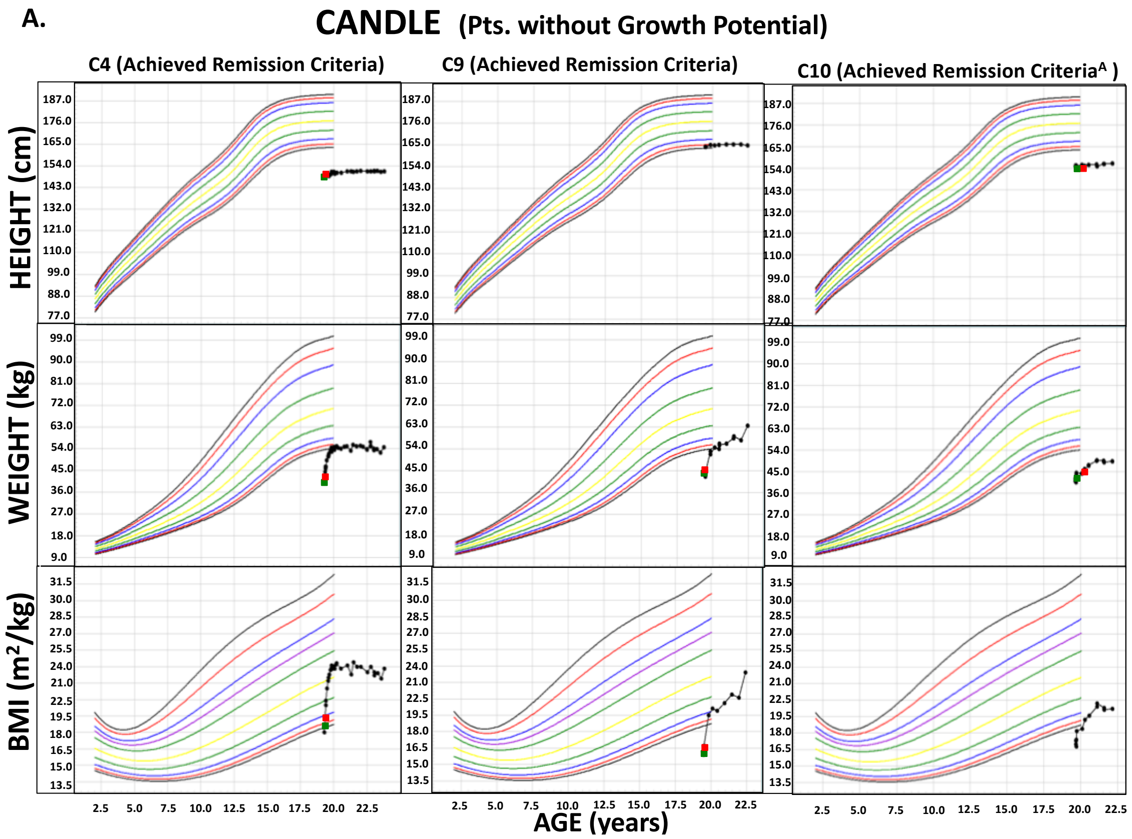

B.

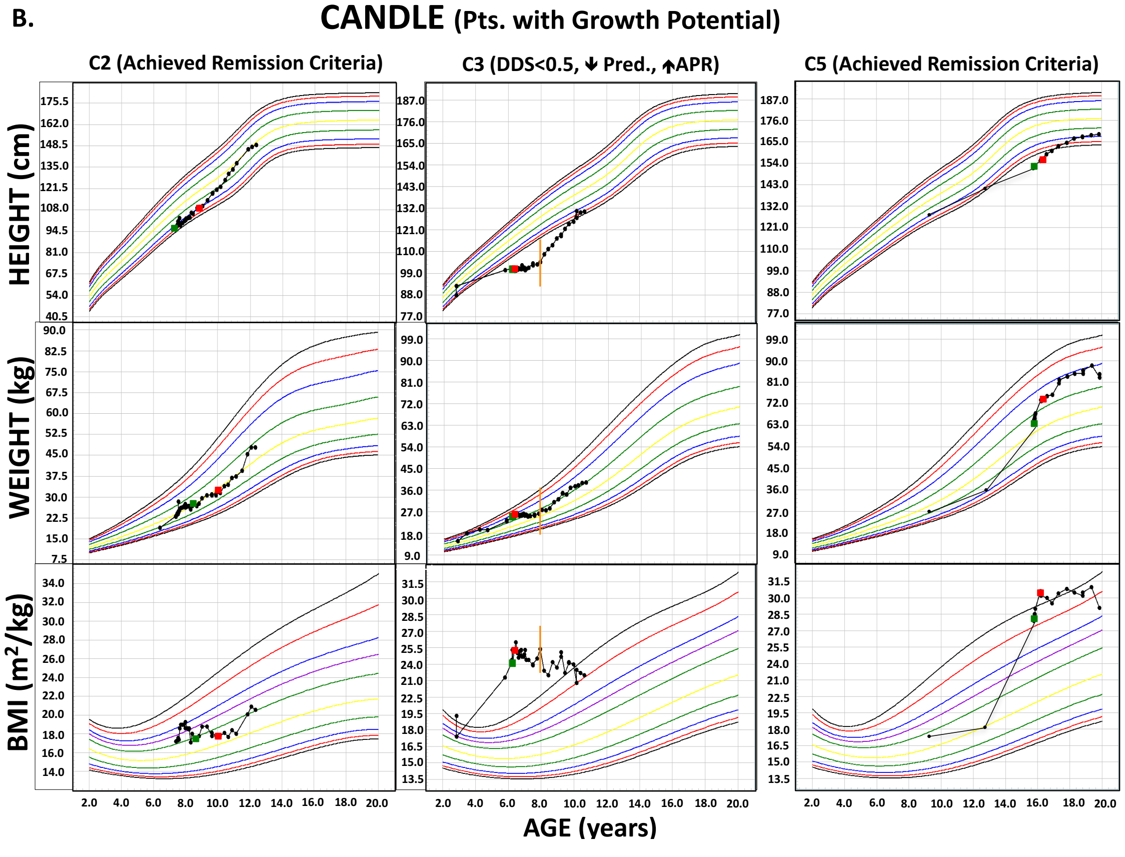

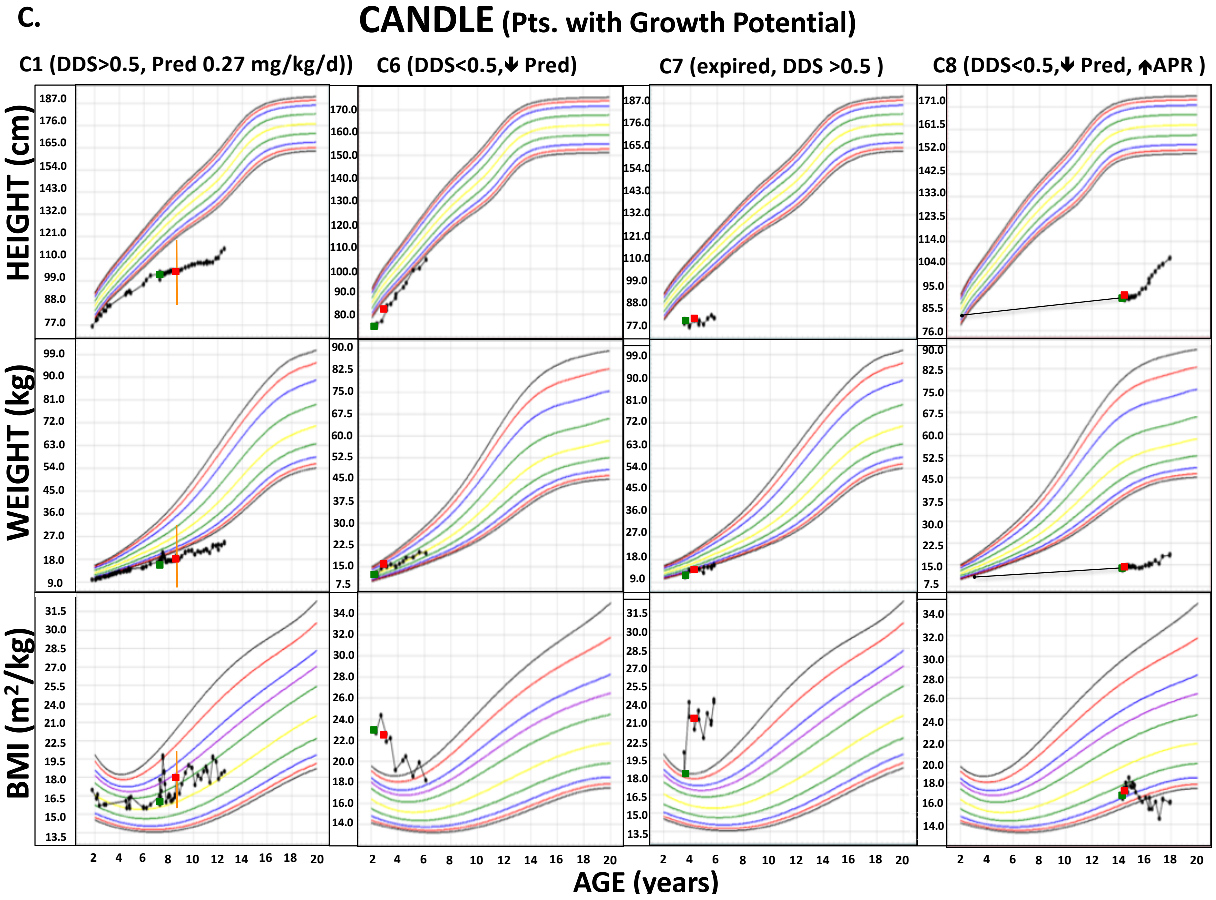
C.
CANDLE (Pts. with Growth Potential)
C1 (DDS>0.5, Pred 0.27 mg/kg/d))
C6 (DDS<0.5,↓ Pred)
C7 (expired, DDS >0.5)
C8 (DDS<0.5,↓ Pred, ↑APR)
HEIGHT (cm)
WEIGHT (kg)
BMI (m²/kg)
AGE (years)

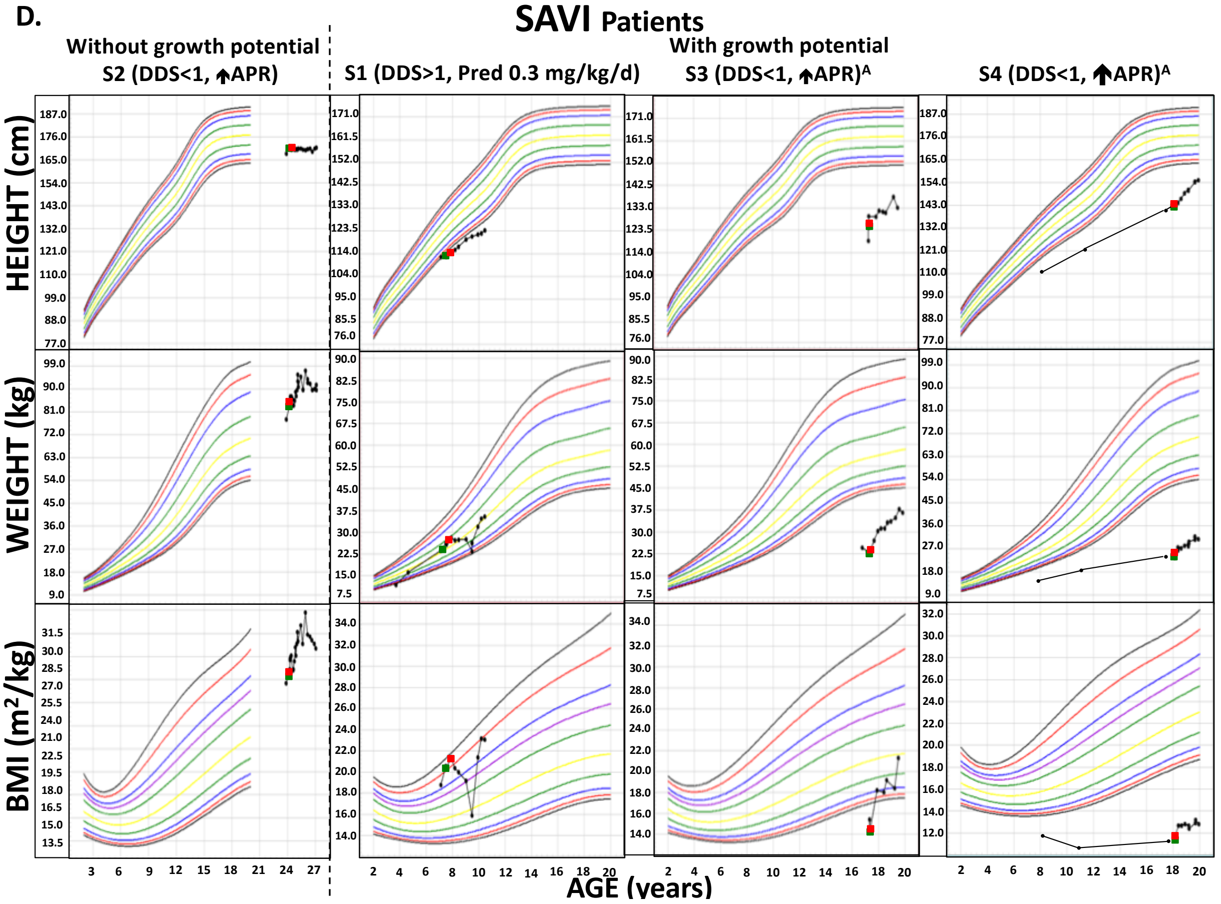
D.
SAVI Patients
Without growth potential
S2 (DDS<1, ↑APR)
With growth potential
S1 (DDS>1, Pred 0.3 mg/kg/d)
S3 (DDS<1, ↑APR)A
S4 (DDS<1, ↑APR)A
HEIGHT (cm)
WEIGHT (kg)
BMI (m²/kg)
AGE (years)

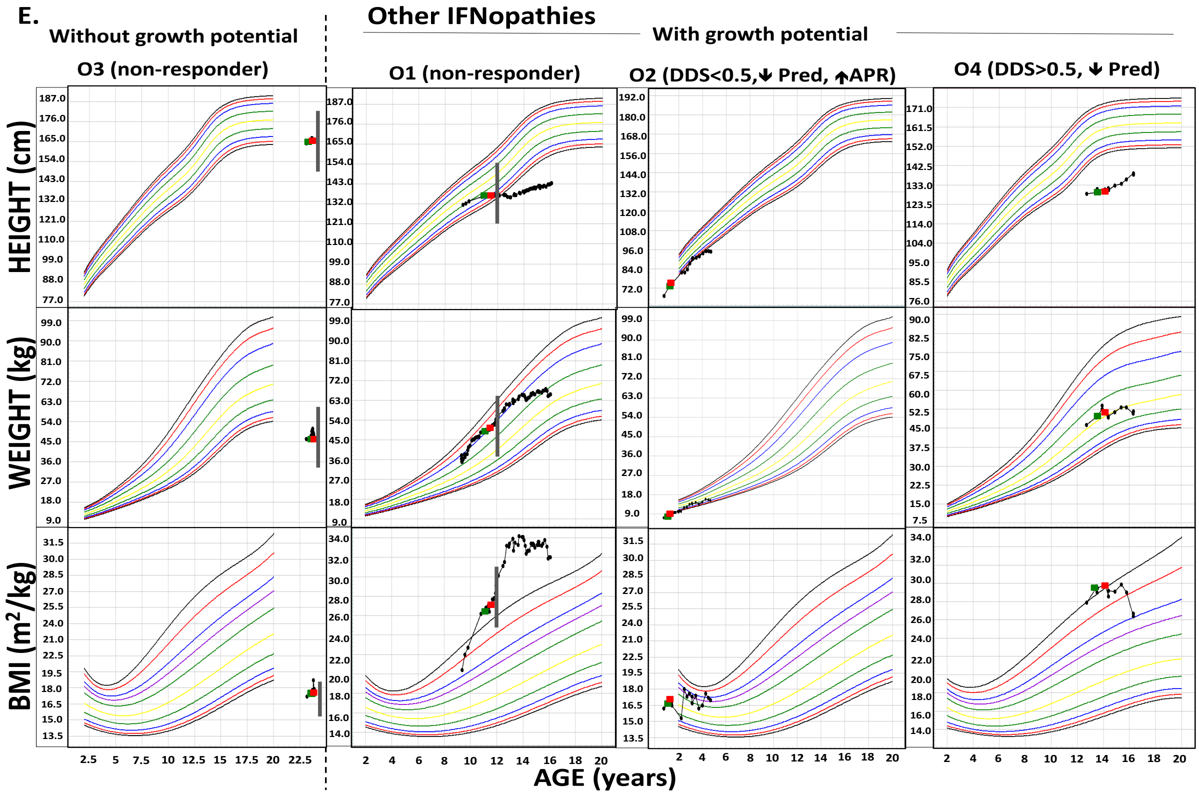

(A) All 3 CANDLE patients (C4, C9 and C10) without growth potential achieved clinical and inflammatory remission criteria. Patients C4 and C10 had weights, heights and body mass indexes (BMIs) below the 3rd percentile, patient C9's height was at the 5th percentile. Upon treatment with baricitinib all 3 patients rapidly gained weight and normalized their BMIs.
[A] CANDLE Patient C10 was not on steroids at the beginning of the program.
(B) Patients C3 and C5 had heights below 3rd percentile at time of entry, upon baricitinib treatment both patients increased their linear growth and normalized BMIs. Patients C2 and C5 achieved criteria for clinical and inflammatory remission. Patient C3 achieved response criteria for reduction of daily diary score (DDS) and prednisone <0.15 mg/kg/day, however continues with elevated acute phase reactants (APR). Patient C3 was started on growth hormone at age of 8 years.
(C) CANDLE patients (C1, C7 and C8) had heights and weights below the 3rd percentile, patient (C6) was also below the 3rd percentile for height, however, her weight was normal. Upon treatment with baricitinib, patients C6 and C8 were able to decrease daily steroids by more than 50% from prednisone dose at baseline. They both showed significant improvement in linear growth and body mass indices. Patient C1 (compound heterozygous for *PSMB4*) remains steroid dependent at doses > than 0.25 mg/kg/day, despite growth hormone initiation, he has a slow improvement in linear height. Patient C7 was discontinued from the program after the development of azotemia and BK viremia. He expired 4 weeks after discontinuation of baricitinib. DDS denotes daily diary score, Pred, prednisone; APR, acute phase reactants.
(D) All SAVI patients except one (S2) were < 18.1 years of age at enrollment and considered to have growth potential. SAVI patients (S3 and S4) had heights and weights below the 3rd percentile, patient (S1) was below 3rd percentile for height but weight was normal. Upon treatment with baricitinib, patients S3 and S4 improved linear growth and body mass indexes, however, both continue to be underweight with BMIs < 18.5. Patient (S1) had an improvement in linear height that correlated with a transient decrease in prednisone. Growth velocity slow down and weight increase, after prednisone dose was increase due to subjective reports of respiratory symptoms (PFTs and Chest CT were stable). Patient S2 continued to gain weight on baricitinib, BMI as of last visit was >30 (obese range). [A] SAVI patients S3 and S4 were not on steroids at the beginning of the program.
(E) Patient O4 (homozygous for *SAMDH1* deletion) and patient O2 (likely novel mutation) were able to decrease steroids and have an improvement in their linear height. Patient O4 normalized her BMI. Patient O1 discontinued from the program due to lack of efficacy, and patient O3 who also had a poor response, developed multifocal avascular necrosis, and was discontinued due to osteonecrosis. DDS denotes daily diary score, Pred, prednisone; APR, acute phase reactants.

Supplemental Figures 5A-C. Metabolic syndrome in patients with CANDLE vs. SAVI

A. Hepatosteatosis at baseline

C5, hypertriglyceridemia, hepatosteatosis and obesity at baseline.

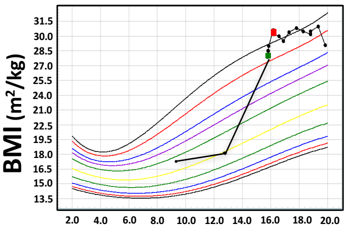

S2, hepatosteatosis (normal triglycerides) and overweight at baseline.

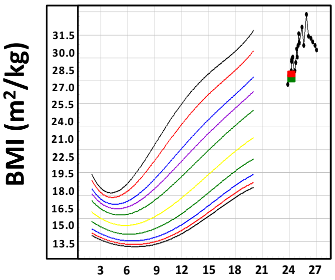

B. Development of hepatosteatosis while on baricitinib

C3, hypertriglyceridemia and obesity at baseline, developed hepatosteatosis. hypertriglyceridemia responded to fenofibrates.

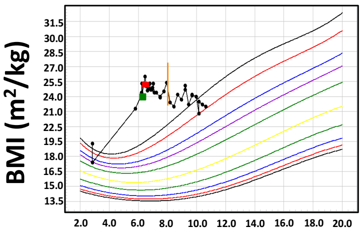

C4, hypertriglyceridemia and borderline low BMI at baseline, after normalization of BMI developed hepatosteatosis. Lack of response to fenofibrates

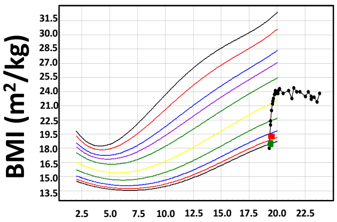

C8, hypercholesterolemia at baseline, developed hepatosteatosis.

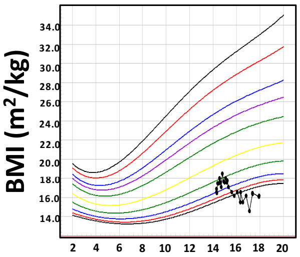

C1, hypertriglyceridemia and normal BMI at baseline, triglycerides improved on fenofibrates without development of hepatosteatosis yet

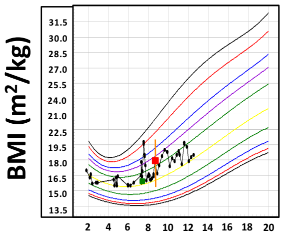

C.

Abdominal MRI CANDLE patient

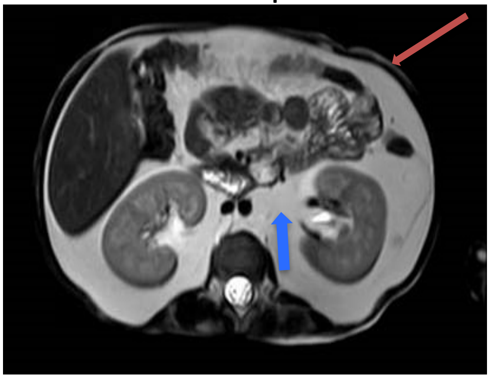

Abdominal MRI SAVI Patient

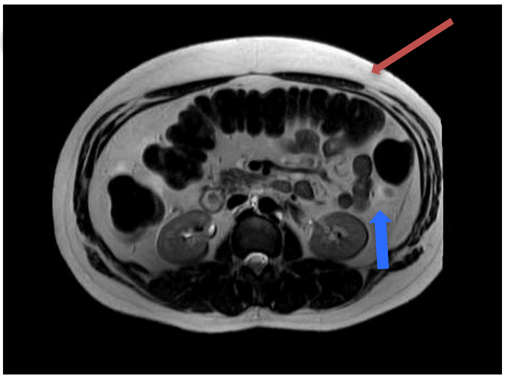

(A) Two patients (C5 and S2) had preexisting hepatic steatosis, documented by magnetic resonance imaging (MRI) within 2 months of baricitinib initiation, C5 with CANDLE was obese had hypercholesterolemia and hypertriglyceridemia, patient S2 with SAVI was overweight with a normal lipid profile. **(B)** Throughout the duration of the program, 3 CANDLE patients (C3, C4 and C8) developed hepatic steatosis, C3 – was obese (>99th percentile), had hypertriglyceridemia and was on treatment with fenofibrates at baseline. On baricitinib treatment, BMI improved but continues to be at the 95th percentile. C4 – had low borderline normal BMI and hypertriglyceridemia at baseline, hepatic steatosis developed with normalization of BMI, C8 – had normal BMI with hypercholesterolemia. All CANDLE patients, except one (Pt. C1), who had hypertriglyceridemia at baseline developed hepatic steatosis on baricitinib treatment. Interestingly, pt. C1, who was started on fenofibrates treatment early but also had a clinical significant reduction of his BMI from the 77th to the 56th percentile, did not develop hepatic steatosis. **(C)** Abdominal MRI in SAVI vs. CANDLE patient: Left: in CANDLE: absence of subcutaneous fat (red arrow) and accumulation of intraabdominal fat (blue arrow) Right: in SAVI: presence of increased subcutaneous fat layer (red arrow) is seen in the context of accumulation of intra-abdominal fat (blue arrow).

Supplemental Figure 6. Cell subsets in CANDLE, SAVI and other IFNopathies

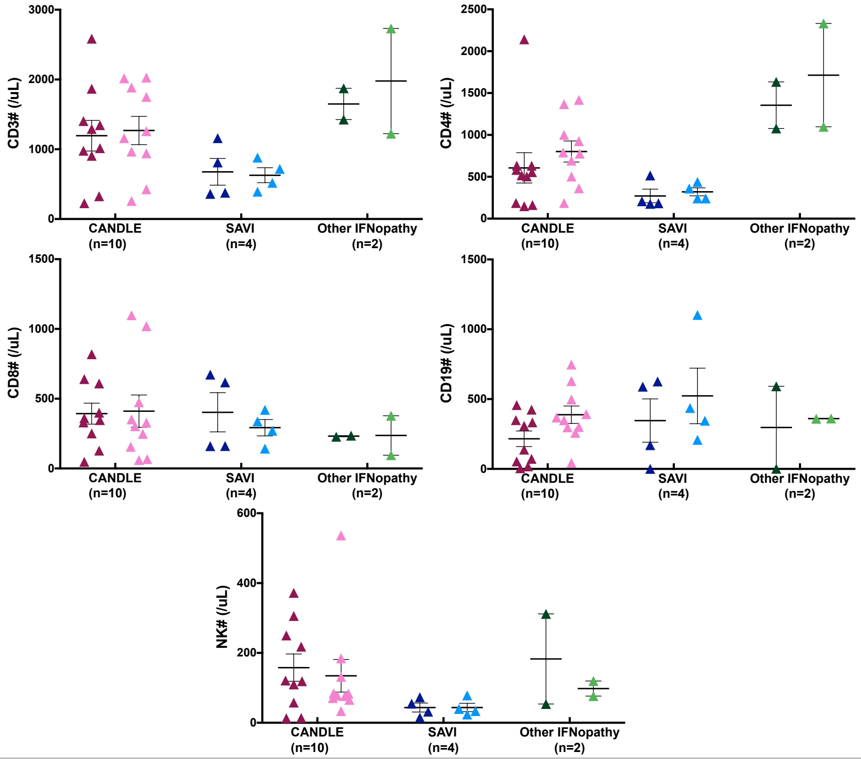

There were no significant changes on TBNK cell subsets in CANDLE, SAVI and patients with other interferon mediated autoinflammatory diseases treated with baricitinib (n=16). Patients O1 and O3 (discontinued from the study) were not included. Data is presented by disease with CANDLE in red, SAVI in blue and other interferonopathies in green. Darker shades indicate pre-treatment and lighter shades last included visit on baricitinib treatment. Means and standard errors are depicted.

Supplemental Figure 7. Quantitative immunoglobulins in CANDLE, SAVI and other IFNopathies

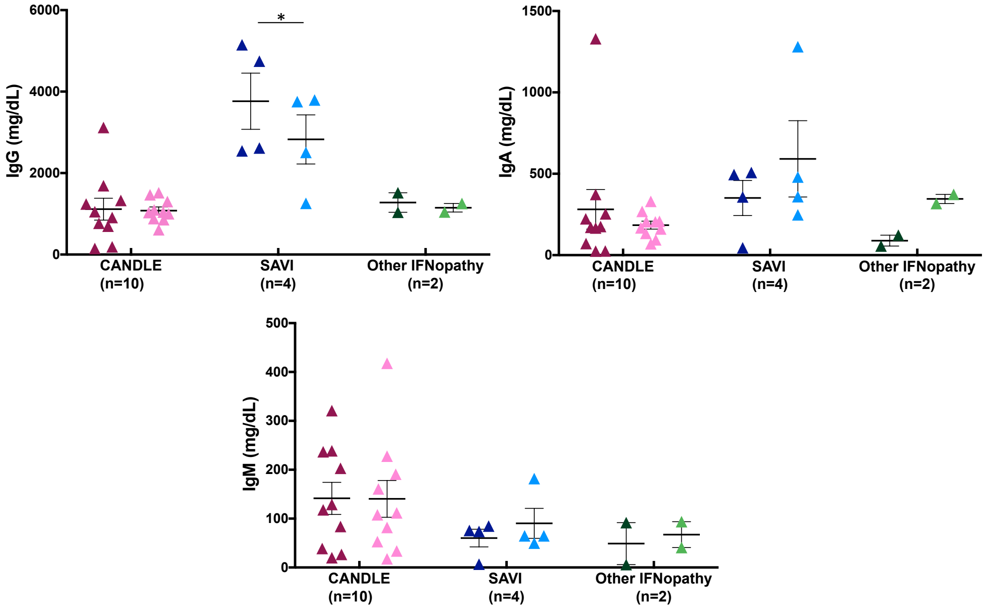

There were no significant changes on quantitative immunoglobulins in CANDLE, SAVI and patients with other interferon mediated autoinflammatory diseases treated with baricitinib (n=16). Patients O1 and O3 (discontinued from the study) are not included. Data is presented by disease with CANDLE in red, SAVI in blue and other interferonopathies in green. Darker shades indicate

pre-treatment and lighter shades last included visit on baricitinib treatment. Means and standard errors graphed.* p-value <0.5

Supplemental Figure 8. Erythrocyte sedimentation rate (ESR) and C-reactive protein (CRP) response to baricitinib

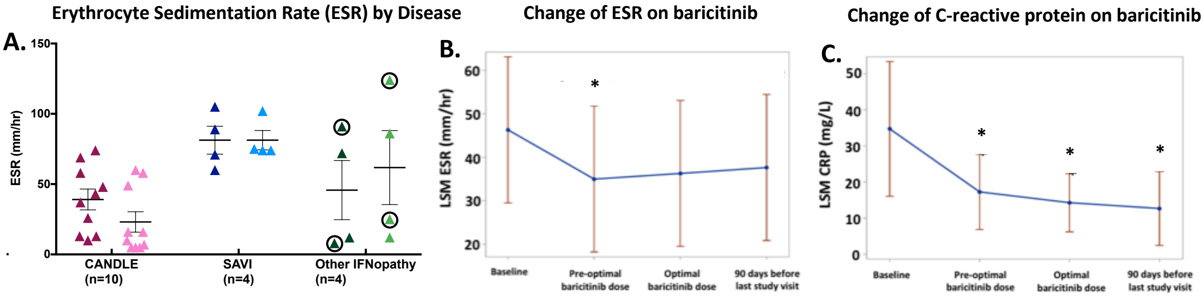

(A) ESR only moderately improved on baricitinib treatement. Despite the clinical responses seen in SAVI patients (i.e. improvement of chronic ulcers and episodes of skin vasculitis and prevention of tissue loss), SAVI patients continue to have an elevated ESR and only 2/4 (50%) had improvement in CRP. The 2 patients who discontinued from the program due to lack of efficacy are circled.

(B, C) Longitudinally assessed CRP, ESR were fitted to a repeated-measures model with "treatment phase" as a categorical independent variable. Least-squares means (LSM) of CRP and ESR with 95% confidence intervals for each phase are graphed. Least-squares means during pre-optimal baricitinib dose, optimal tolerated baricitinib dose, and 90 days before last visit were compared to baseline least-squares means.

* Denotes unadjusted *p-values < 0.05*

Supplemental Figure 9. Evaluation of biomarkers of IFN signaling and correlation with daily symptoms and ability to wean corticosteroids.

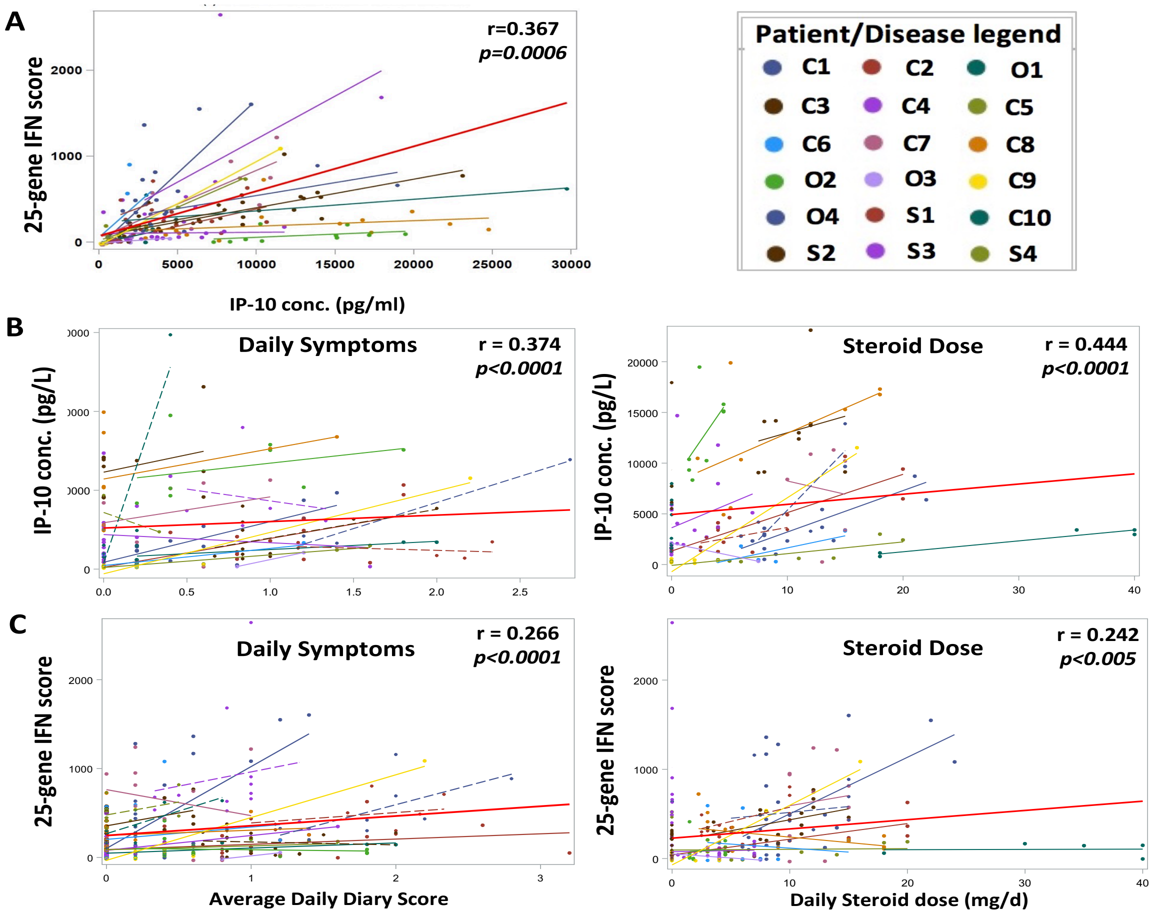

(A) Serum IP-10 levels and the 25-gene IFN score significantly correlate. The r-values and p-values are indicated in the left upper corner. **(B, C)** The IP-10 serum concentration and the 25-gene IFN scores respectively correlate with improvement in daily symptoms and daily corticosteroid dose. The r-values and p-values are indicated in the right upper corner.

Supplemental Figures 10A,B. Evaluation of biomarkers of IFN signaling

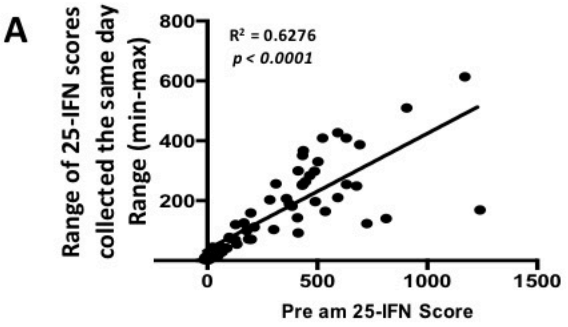

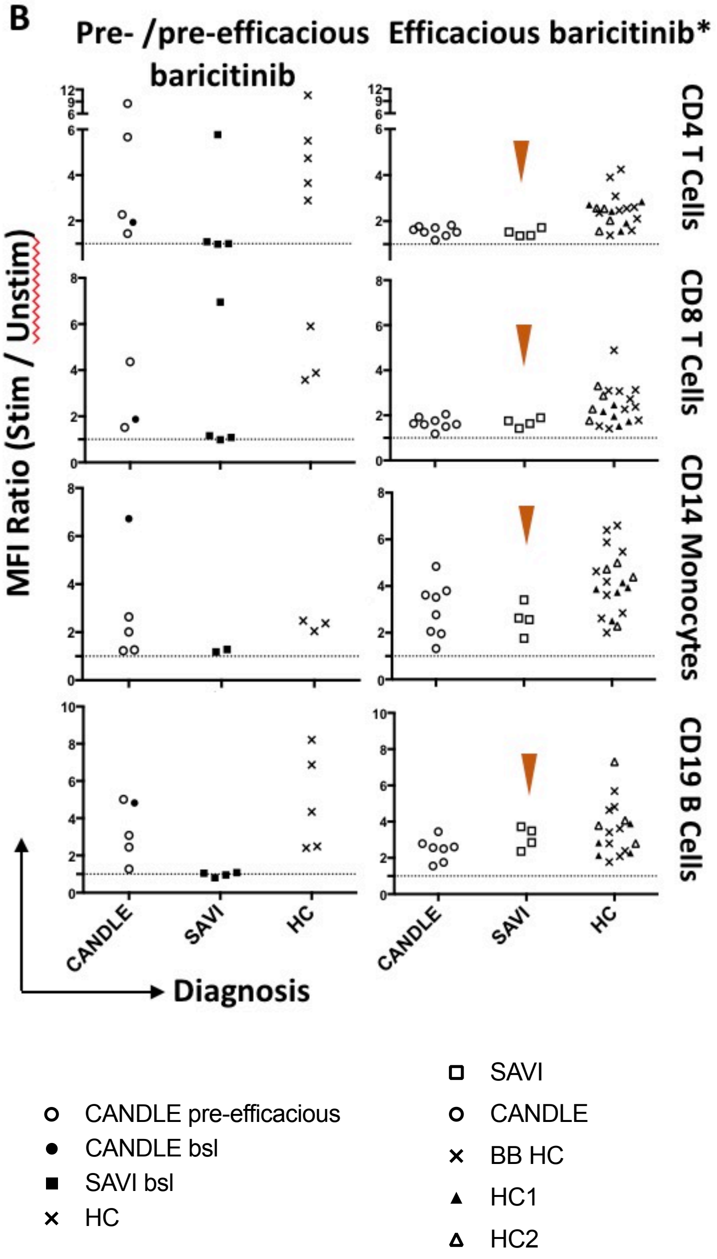

(A) RNA samples collected from 10 CANDLE, 4 SAVI and 3 other interferonopathy patients at multiple timepoints during the same day were assessed for the 25-gene IFN score. Linear regression of the range of IFN scores over a day versus the IFN score of the first morning sample (time 0) is significantly linear with higher range or variability noted at higher scores. Equation: Y = 0.387222*X + 37.35 with an R^2 =0.6276 and a *p-value<0.0001*.

(B) STAT1 Phosphorylation with IFNα stimulation before baricitinib treatment (historical data) or on low pre-optimal doses of baricitinib (left panels) compared to optimal tolerated baricitinib doses (right panels). As previously reported (Liu et al. NEJM 2014), STAT-1 phosphorylation in SAVI patients before treatment with baricitinib was maximally up-regulated and was not further induced with IFN stimulation (MFI ratios of stimulated over unstimulated cells equaled 1). Post baricitinib, STAT-1 phosphorylation was assessed in 8 CANDLE and 4 SAVI patients. Blood samples were obtained in the morning, before baricitinib dose administration (at the baricitinib trough level). Two healthy controls with repeated measurements (HC1, HC2) and healthy controls with one blood draw only (n=10) were compared with CANDLE (n=8) and SAVI (n=4) patients' samples. (Panel C (right panel) is modified from Kim et al. Clin Pharmacol Ther 2017. Red arrowheads indicate increase in MFI ratio in SAVI patients on treatment which is up compared to pre-baricitnib (left panels). *patients were on optimal tolerated doses of baricitinib at the time of blood draw.

As different methods for the STAT phosphorylation were used, no formal statistical comparisons between pre-/baricitinib of per-efficacious doses of baricitinib and optimal doses of baricitinib were performed.

Supplemental Figure 11. Cytokine changes on baricitinib

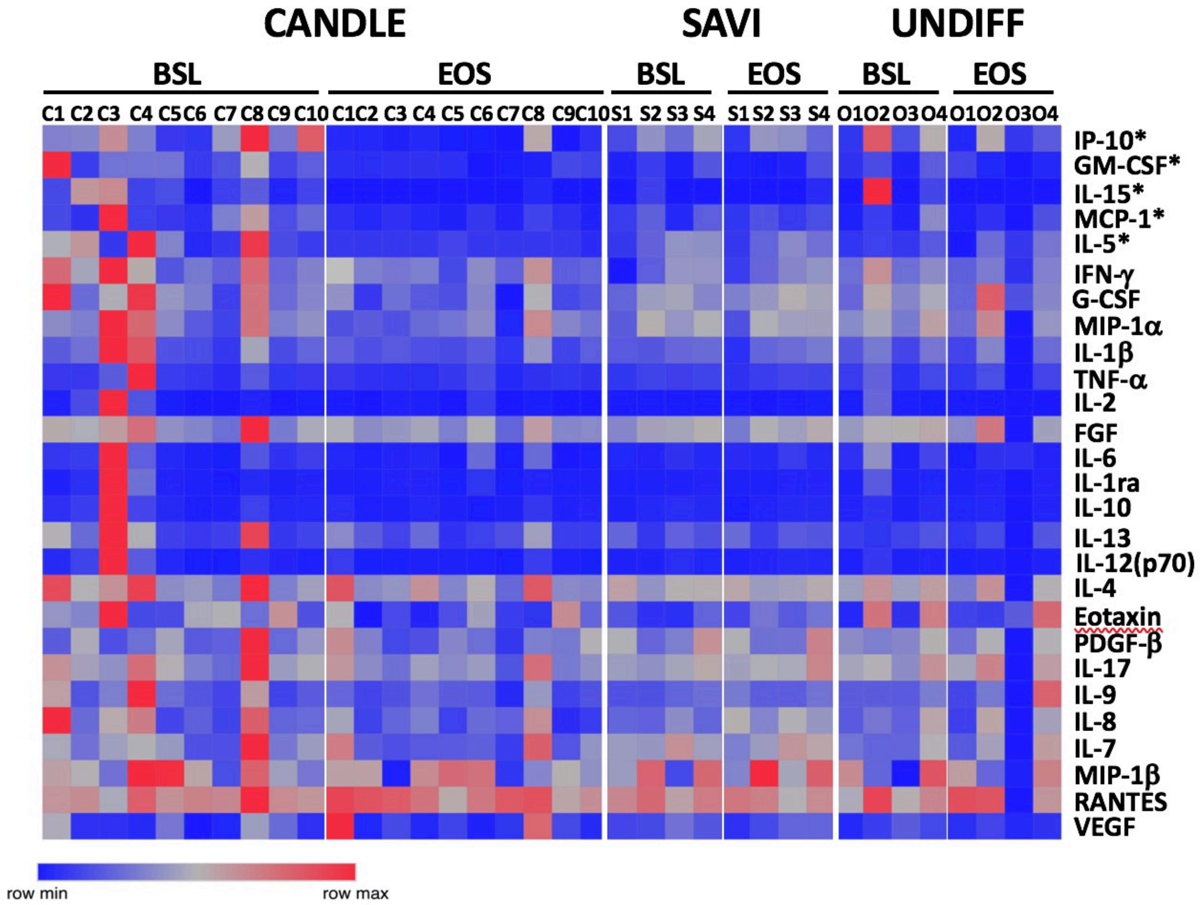

CYTOKINE	CANDLE baseline	CANDLE EOS	p-value	SAVI baseline	SAVI EOS	p-value2	UNDIFF baseline	UNDIFF EOS	p-value3
**IP-10**	8449.4±5697.5	1631.5±3135.6	***0.0005***	6231.5±2594.3	5459±2876.4	0.6154	7813.0±6569.5	4354.9±4246.1	0.083
**GM-CSF**	176.5±147.7	41.2±26.5	***0.0204***	54.3±46.1	36.6±43.5	0.2715	71.4±47.7	35.4±32.0	0.305
**IL-15**	78.3±77.3	4.4±4.4	***0.0146***	13.2±20.5	7.4±8.8	0.6707	101.6±166.2	3.0±0.0	0.321
**MCP-1**	535.0±449.0	194.3±51.0	***0.0351***	330.7±136.9	308.2±70.4	0.6115	304.2±247.4	208.5±121.3	0.235
**IL-5**	3.9±3.6	1.0±0.2	***0.0286***	2.8±1.4	2.5±1.1	0.2754	1.3±0.5	1.7±1.4	0.477
**IFN-γ**	423.4±207.1	295.2±101.0	0.0990	256.2±145.1	270.5±95.0	0.6289	353.5±123.8	242.6±90.2	0.107
**G-CSF**	340.2±106.2	248.3±56.1	***0.0049***	299.8±36.7	321.7±28.9	0.1285	311.6±48.3	309.5±109.0	0.958
**MIP-1α**	0.5±0.2	0.4±0.1	0.0773	0.4±0.1	0.4±0.1	0.4604	0.5±0.1	0.4±0.2	0.429
**IL-1β**	2.7±1.7	1.8±0.5	0.1233	2.0±0.2	1.9±0.6	0.8968	2.0±0.4	1.7±0.9	0.288
**TNF-α**	130.1±139.0	67.5±17.6	0.2055	85.2±11.2	85.3±17.0	0.9802	98.3±41.5	74.9±40.1	0.076
**IL-2**	334.8±745.8	39.6±79.5	0.2428	10.3±4.2	12.2±9.1	0.7746	170.0±292.8	8.5±9.5	0.343
**FGF basic**	364.4±90.8	322.1±40.6	0.0730	336.3±28.2	337.6±39.8	0.8862	358.1±23.1	312.0±125.6	0.518
**IL-6**	222.6±286.6	107.1±101.6	0.2724	85.0±20.9	101.5±30.2	0.0614	193.8±160.6	87.1±32.6	0.250
**IL-1ra**	3084±6194.3	620.0±425.6	0.2385	567.9±123.1	610.7±81.0	0.3342	1530.7±1861.9	492.0±250.5	0.320
**IL-10**	82.0±154.5	17.9±12.4	0.2279	21.6±7.8	17.1±10.5	0.1508	24.0±13.5	13.4±8.2	0.096
**IL-13**	12.2±10.3	7.1±3.6	0.0998	6.7±2.1	6.9±1.9	0.6513	4.9±1.4	4.3±2.5	0.656
**IL-12(p70)**	359.1±698.8	82.9±86.5	0.2560	89.3±37.2	84.1±25.3	0.6866	97.8±81.8	44.2±25.6	0.206
**IL-4**	12.9±3.3	11.8±2.8	***0.0355***	12.0±1.0	11.8±0.8	0.5799	12.2±1.2	10.1±3.6	0.211
**Eotaxin**	201.8±89.2	144.1±74.0	0.0981	111.9±29.1	142.5±25.5	0.2179	194.2±127.5	168.4±106.1	0.699
**PDGF-β**	4356.7±2611.8	3784.3±1753.9	0.5500	4892.5±1734.6	5004.8±1970.1	0.6406	4574.1±858.6	3835.3±2489.0	0.565
**IL-17**	343.7±102.6	293.0±77.1	***0.0100***	313.1±31.7	344.8±47.8	0.0991	329.3±47.7	299.7±143.4	0.631
**IL-9**	183.2±93.1	128.5±37.8	***0.0347***	133.9±18.0	149.7±18.2	0.2929	142.7±20.4	169.4±131.1	0.667
**IL-8**	156.7±59.4	124.1±34.8	***0.0198***	127.6±10.1	160.4±15.0	***0.0180***	137.2±30.6	129.3±51.6	0.726
**IL-7**	50.4±23.9	40.6±24.2	0.0824	56.3±10.1	56.6±12.8	0.9561	40.5±13.0	39.5±26.1	0.926
**MIP-1β**	137.7±46.6	122.5±43.6	0.1990	135.3±52.8	155.6±52.3	0.2710	118.0±65.6	108.9±52.2	0.296
**RANTES**	9489.2±1419.0	10176±1313.7	0.2518	9476.9±905.0	9151.7±951.7	0.3939	9135.8±1889.6	8543.3±4043.2	0.769
**VEGF**	178.3±153.0	235.8±298.7	0.3383	191.8±58.0	176.3±70.6	0.5314	138.9±61.3	91.8±32.9	0.321

TOP: The heatmap depicts serum cytokine levels at baseline (BSL) and at the last visit (end of study, EOS). Samples were collected up to December 2015. The cytokines are sorted in the heatmap according to the level of statistical significance of the BSL vs. EOS comparisons and cytokines marked with an asterisk were significantly higher at baseline. The reduction of serum IP-10 levels, a downstream marker of IFN signaling was most pronounced. Other serum cytokine levels that significantly dropped on treatment included the myeloid growth and differentiation factor, granulocyte-macrophage colony-stimulating factor (GM-CSF), and the GM-CSF induced chemokine MCP-1 (CCL2) that is dependent on JAK2-STAT5 signaling through the GM-CSF receptor.[31] Both modulate monocyte and macrophage differentiation[30] and lead to a proinflammatory environment. The treatment effect on the reduction of serum levels of the proinflammatory cytokine, IL-15, that is secreted by mononuclear phagocytes (and some other cells), and the eosinophil and B cell growth and differentiation factor, IL-5, were more pronounced than the effect on IL-6, which was not statistically significant. IL-15 and IL-5 signal through their respective receptors through recruiting JAK2-STATs, and amplify their production through an autocrine loop, IFNα and IFNβ could not be reliably measured.

BOTTOM: The table depicts the comparisons of mean and standard deviations of cytokine serum levels between baseline and end of study (EOS) visit for each group of diseases (CANDLE, SAVI and undifferentiated interferonopathies, UNDIFF), separately.
